# Retinoic acid, an essential component of the roof plate organizer, promotes the spatiotemporal segregation of dorsal neural fates

**DOI:** 10.1242/dev.202973

**Published:** 2024-09-30

**Authors:** Dina Rekler, Shai Ofek, Sarah Kagan, Gilgi Friedlander, Chaya Kalcheim

**Affiliations:** ^1^ Department of Medical Neurobiology, Institute of Medical Research Israel-Canada (IMRIC) and the Edmond and Lily Safra Center for Brain Sciences (ELSC), Hebrew University of Jerusalem-Hadassah Medical School, Jerusalem 9112102, Israel; ^2^ The Mantoux Bioinformatics Institute of the Nancy and Stephen Grand Israel National Center for Personalized Medicine, Weizmann Institute of Science, Rehovot 7610001, Israel

**Keywords:** BMP, Cell specification, Dorsal interneurons, Dorsal root ganglia, Melanocytes, Neural crest, Neural tube, Notch, Roof plate, Single-cell RNA sequencing, Quail

## Abstract

Dorsal neural tube-derived retinoic acid promotes the end of neural crest production and transition into a definitive roof plate. Here, we analyze how this impacts the segregation of central and peripheral lineages, a process essential for tissue patterning and function. Localized *in ovo* inhibition in quail embryos of retinoic acid activity followed by single-cell transcriptomics unraveled a comprehensive list of differentially expressed genes relevant to these processes. Importantly, progenitors co-expressed neural crest, roof plate and dI1 interneuron markers, indicating a failure in proper lineage segregation. Furthermore, separation between roof plate and dI1 interneurons is mediated by Notch activity downstream of retinoic acid, highlighting their crucial role in establishing the roof plate–dI1 boundary. Within the peripheral branch, where absence of retinoic acid resulted in neural crest production and emigration extending into the roof plate stage, sensory progenitors failed to separate from melanocytes, leading to formation of a common glia-melanocyte cell with aberrant migratory patterns. In summary, the implementation of single-cell RNA sequencing facilitated the discovery and characterization of a molecular mechanism responsible for the segregation of dorsal neural fates during development.

## Teaser

**Summary:** Timely segregation of specific central and peripheral neural fates in quail depends on the concerted activity of a retinoic acid, BMP, Wnt and Notch signaling network.

## INTRODUCTION

The separation of central and peripheral branches of the nervous system (CNS and PNS, respectively), takes place in the dorsal neural tube (NT), offering a prototypic example of lineage segregation during development. This domain sequentially generates neural crest cells (NCs), progenitors of the PNS, followed by the definitive roof plate (RP) of the CNS, which is bordered ventrally by dorsal interneurons (Chizhikov and Millen, 2005; Kalcheim and Rekler, 2022; Rekler and Kalcheim, 2021).

NC progenitors undergo a bone morphogenetic protein (BMP)-dependent epithelial-to-mesenchymal transition (EMT), exit the NT and generate neurons and glia of peripheral ganglia, as well as melanocytes (Bronner, 2012; Le Douarin and Kalcheim, 1999; Sela-Donenfeld and Kalcheim, 1999). RP progenitors originate ventral to the premigratory NCs, relocate ventro-dorsally as a result of continuous NC emigration, and reach the midline of the NT upon completion of NC departure (Krispin et al., 2010a,b). In contrast to NCs, RP cells change their state of competence and become refractory to BMP despite continuous ligand synthesis (Nitzan et al., 2016). They progressively exit the cell cycle and act as a dorsal organizer, providing the BMP and Wnt ligands required for interneuron development (Augsburger et al., 1999; Chizhikov and Millen, 2005; Gupta et al., 2022; Kahane and Kalcheim, 1998; Lee et al., 2000; Nitzan et al., 2016; Phan et al., 2010).

Hence, our understanding of cellular states/fates and the mechanisms of transitions progressively evolves. However, the identification of mechanisms responsible for lineage separation of NC-derived cell types, of NCs from RP cells, and RP cells from adjacent dorsal interneurons, remains challenging at the experimental level, given the rapid temporal dynamics of these processes.

A prerequisite for tackling the transition between NCs and RP cells is to know how NC production ceases. We previously found that dorsal NT-derived retinoic acid (RA) ends the period of NC production and emigration via inhibition of BMP/Wnt activities. This is achieved by restricted and dynamic expression of BMP antagonists in the dorsal NT and by a network of specific downstream effectors. In the absence of RA activity, NC emigration extends into the RP period, and dI1 interneurons also invade the RP territory. Hence, the temporal and spatial segregation of dorsal lineages, as well as the structural integrity of the RP, are abnormal in these conditions (Rekler and Kalcheim, 2022). Furthermore, both gain and loss of Notch function alter the balance between RP and dI1 interneurons, suggesting that this pathway is required for setting the RP–dI1 boundary (Ofek et al., 2021). A refined analysis of the above processes and their integration into a comprehensive molecular network are still lacking. To approach these important questions, and in particular, the involvement of RA in fate segregation of both central and peripheral neural progenitors, a single-cell approach is required.

By inhibiting RA activity locally followed by single-cell RNA-seq we here uncover: first, a large set of differentially expressed genes compared with normal embryos; second, a lack of RA prevents the separation of NC, RP and dI1 interneuron fates, leading to combinatorial co-expression of lineage markers within single cells; and third, Notch activity downstream of RA mediates the separation between RP and dI1 interneurons.

Moreover, in the absence of RA, the extended production and emigration of NCs led to PNS progeny in which separation of sensory glia and melanocytes, thought to derive from a common progenitor (Colombo et al., 2022; Dupin et al., 2000, 2003; Real et al., 2006), was hindered. Consequently, single cells with glia-melanocyte traits were apparent. These findings show that RA is responsible for the segregation of numerous dorsal neural fates in a cell type-specific manner, highlighting the significance of RA and related signaling pathways in normal fate acquisition and tissue patterning.

## RESULTS

### Single-cell transcriptomics identifies the developmental dynamics of cell types in the dorsal NT

**Fig. 1.**
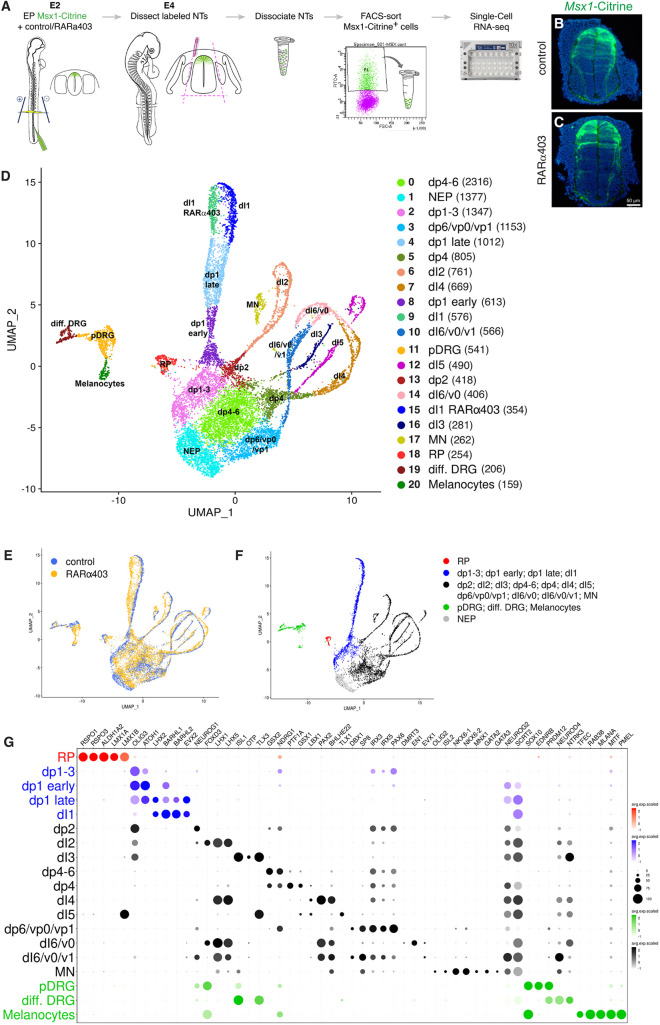
**Assignment of transcriptomes to cell identities.** (A) Experimental design for isolating single dorsal NT cells. (B,C) NTs electroporated at E2.5 and microdissected at E4, showing similar Msx1-Citrine labeling between control and RARα403 treatments. (D) UMAP of control and treated samples, colored by cluster with cell count indicated. (E) UMAP of entire dataset colored by sample. (F-G) Cluster identification based on marker gene expression in control sample. Dot plot (G) shows average expression (color depth) and percentage of expressing cells per gene (circle size). dI, dorsal interneurons; diff. DRG, differentiated dorsal root ganglion neurons; dp, dorsal interneuron progenitors; EP, electroporation; FACS, fluorescence-activated cell sorting; MN, motoneurons; NEP, neuroepithelial progenitors; NT, neural tube; pDRG, dorsal root ganglion progenitors; RP, roof plate. Scale bar: 50 µm.

To enrich for dorsal NT cell types, quail embryonic day (E) 2.5 NTs were electroporated in the flank region with Msx1-Citrine (Williams et al., 2019) combined with either a control vector, or RARα403, a dominant-negative RA receptor. For each group, ten NTs with associated mesoderm were mechanically microdissected at E4 and single-cell suspensions prepared. Fluorescent cells were then sorted and subjected to droplet-based single-cell RNA sequencing (scRNA-seq) (10x Genomics Chromium) to generate a gene expression profile of control and treated dorsal NT cells (Fig. 1A-C). In total, 17,549 cells were sequenced. After applying quality filters (see Materials and Methods), a dataset of 14,566 cells was retained for further analysis (5890 control and 8676 treated cells, which comprise 86.6% and 80.7% of the initial number of cells, respectively).

#### Assignment of transcriptomes to cell identities

Unsupervised clustering identified 21 cell clusters, visualized using uniform manifold approximation and projection (UMAP) (Fig. 1D, Table S1). Most clusters contained similar numbers of control and treated cells (Fig. 1E, Fig. S1A). The clusters were allocated to different cell types based on combinatorial expression of a selected list of established genes in the control sample (Fig. 1F,G) (Delile et al., 2019; Faure et al., 2020). Mainly dorsal NT cell types (RP, dI1-6 interneurons) and NC-derived dorsal root ganglia (DRG) and melanocytes (Sharma et al., 1995) were identified, but not NC derivatives bearing a more ventral character.

The latter is consistent with the timing of electroporation that attained only the later emigrating NC progenitors (Krispin et al., 2010b). Clusters 3 and 14 comprised, respectively, progenitors or neurons expressing mixed dI6/v0/v1 characteristics, perhaps owing to poorly resolved transcriptomes, and cluster 17 contained motoneurons. Although significant enrichment of dorsal NT cells was apparent, the presence of various ventral cell types suggests some leaky expression of Msx1-Citrine at early transfection.

#### A map of time and space

The resulting UMAP bears the shape of an ‘octopus-like’ structure with a head and emerging arms. Cell cycle analysis showed that the octopus head (clusters 0, 1, 2, 3) is mainly composed of immature proliferating neuroepithelial progenitors encountered either in G2/M or S phases of the cell cycle (Fig. S1A). Consistent with this, cell cycle-associated genes such as *BUB1* and *CDK1* (Fig. S1C), and the neural progenitor marker *SOX2*, for example, were expressed. Whereas cluster 1 was composed of neuroepithelial progenitors (NEPs) lacking a specific identity, clusters 0, 2 and 3 contained defined dorsal interneuron precursors. Clusters 8, 13 and 5, connecting the head of the octopus with its arms, exhibited specific expression of early neuronal progenitor genes characterized by the Notch ligands *DLL1* and *JAG2* (Fig. S1C). Furthermore, the neuronal specification markers *NEUROD1* and *NEUROG2* decorated the proximal portions of all arms, including young E4 DRG neurons (cluster 19) (Fig. S1C). Finally, the distal portions of the arms expressed neuronal markers such as *ELAVL2*, *EBF1*, contactin 2 (*CNTN2*), *ROBO3* (Fig. S1C), as well as *NEFM*, *NTRK3*, *NHLH2*, *SEPTIN3*, *ONECUT1* and -*2*, etc. A majority of cells in the latter category were in the G1 phase of the cell cycle (Fig. S1B,C). Thus, the head-to-arm dimension reflects a temporal sequence of neural progenitor differentiation, and highlights the co-existence of immature precursors and differentiated neurons in the E4 NT.

Likewise, a spatial map of cell types was deduced along the left-to-right direction of the UMAP (Fig. S1C,D), with dorsal cell types expressing *FOXD3* in DRG, *RSPO1* in RP cells; *WNT1* in RP cells and dp1-3 progenitors; *OLIG3* in dp1-3 cells, dI2 and dI3; *GSX2* in dp4-6; graded *LGR5* in dp6 to dp4-6; *LHX2* in dI1; *FOXD3* in dI2; *TLX3* in dI3/5; and *PAX2* in dI4/6 (Fig. S1E). Hence, the left-to-right dimension of the UMAP corresponds to a dorsoventral distribution of cell types in the E4 NT.

### RA signaling prompts fate segregation of dorsal NT cell types

#### Analysis of the normal RP predicts gene expression patterns

Next, we focused on the development of the RP. Fig. S2 illustrates the profile of the most significant genes expressed in control RP cells compared with remaining dorsal clusters. In addition to confirmation of the presence of previously studied RP-specific genes, e.g. *LMX1A*, *LMX1B* (Chizhikov and Millen, 2004a,b), *GDF7* (Lee et al., 1998), *BMP4*, *BMP5*, *BMP7*, *BAMBI*, *ALDH1A2*, *WNT1* and *WNT3A* (Krispin et al., 2010a; Ofek et al., 2021; Rekler and Kalcheim, 2022), the presence of further genes was also identified. These include *LMO7*, olfactomedin3 (*OLFML3*), calponin 2 (*CNN2*), chondromodulin (*CNMD*), *CD99*, *CD82*, *ADAMTS8*, *ADAMTS3*, *WNT9A*, *UNC5C*, *TM6SF1*, *SPON1*, *RSPO3*, *KCNIP4*. An additional array of mRNAs, relatively enriched in RP cells vis-à-vis dorsal interneurons and/or their progenitors (e.g. *LOXL1*, *LOXL3*, *LAMC1*, *CTBP2*, *DRAXIN*, *ZIC1-3*, etc.), was also identified. Together, these newly identified RP genes provide a valuable resource for further functional analysis.

#### Differential gene expression in the RP of control and RARα403-treated NTs

**Fig. 2.**
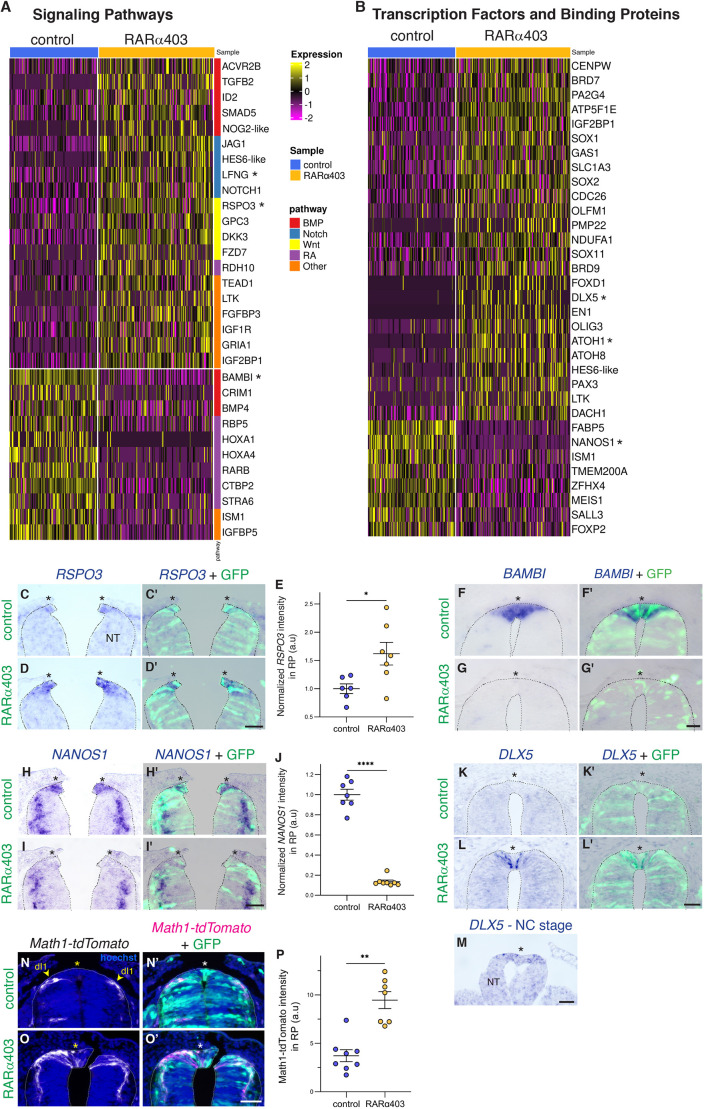
**Differential gene expression in the RP reveals changes in signaling pathways and transcription factors.** (A,B) Heatmaps of differentially expressed genes in response to RARα403 treatment in the RP cluster. Shown are selected genes with a minimum linear fold change of ±1.3 and adjusted *P*-value <0.05. Signaling pathway genes are depicted in A and transcription factors and binding protein categories in B. Genes validated *in vivo* are marked with an asterisk. (C-M) ISH on embryos electroporated with GFP along with control PCAGG or RARα403 at E2.5 and analyzed at E4. The RP is marked by asterisks. (C-E) ISH for *RSPO3*, showing upregulation in treated embryos. *n*=6,7 embryos for control and treated groups, respectively. (F-G′) ISH for *BAMBI*, showing downregulation in treated embryos (see Rekler and Kalcheim, 2022 for details). (H-J) ISH for *NANOS1*, showing downregulation in the RP of treated embryos. *n*=7,8 embryos for control and treated groups, respectively. (K-M) *DLX5* mRNA is expressed in premigratory NCs (M), downregulated in control RP (K,K′), yet shows extended expression in treated RP (L,L′). *n*=6,6,3 for control, RARα403 and NC groups, respectively. (N-P) Electroporation at E2.5 with GFP and Atoh1-tdTomato, along with control PCAGG or RARα403, followed by fixation at E4. The Atoh1-tdTomato reporter labels dI1 cell bodies and processes. Yellow arrowheads mark the location of dI1 cells ventral to RP in control embryos. Note, however, invasion of the treated RP by cells with reporter activity. *n*=8,7 embryos for control and RARα403 groups, respectively. a.u., arbitrary units; dI1, dorsal interneurons1; NC, neural crest; NT, neural tube; RP, roof plate. *P<0.05, **P<0.005, ****P<0.0001, Welch's *t*-test (E,J) and Mann–Whitney test (P). See Table S6 for source data. Scale bars: 50 µm.

Next, we addressed the effects of inhibiting RA signaling on RP development. Changes in gene expression between control and RARα403-treated samples revealed profound effects in four main categories: (1) signaling pathways; (2) transcription factors and binding proteins; (3) extracellular matrix (ECM), EMT and migration; and (4) axonal growth and guidance. Validation of representative genes by *in situ* hybridization (ISH) and reporter assays confirmed the bioinformatic data (Fig. 2, asterisks).

As previously reported, BMP and Wnt activities are maintained in RA-depleted dorsal NTs as opposed to their normal downregulation in the RP (Rekler and Kalcheim, 2022). Consistent with this, numerous Wnt pathway genes were upregulated, including *RSPO3*, and so were the positive BMP effectors *ID2* and *SMAD5* (Fig. 2A) (Das and Crump, 2012; Monteiro et al., 2008). Simultaneously, BMP inhibitors such as *BAMBI* and *CRIM1* (Onichtchouk et al., 1999; Wilkinson et al., 2003), as well as *BMP4* itself, were reduced. Together, a balance between positive and negative BMP pathway genes is likely to account for the measured upregulation in factor activity (Nitzan et al., 2016; Rekler and Kalcheim, 2022).

Alterations in RA pathway genes were anticipated upon repression of RA signaling. Indeed, this negatively impacted the RA receptor *RARB*, the retinol binding protein *RBP5*, *STRA6* (Reijntjes et al., 2010) and *CTBP2* (Bajpe et al., 2013), although we observed an upregulation of *RDH10* (Reijntjes et al., 2010). Notably, all genes related to the Notch pathway were upregulated, hinting at a possible repression of RA on Notch signaling (Fig. 2A).

Many transcription factors were affected in RARα403-treated NTs (Fig. 2B). These included upregulated *SOX2*, consistent with RA acting as a differentiation factor in the absence of which cells preserve a progenitor-like state. Notably, the RNA-binding protein *NANOS1* was downregulated (Fig. 2B,H-J). NANOS1 is involved in a variety of processes (De Keuckelaere et al., 2018; Maschi et al., 2023), but its function in the RP is unknown.

Most importantly, a distinct set of upregulated transcription factors consisted of NC- (e.g. *DLX5*) and dI1-specific (*OLIG3*, *ATOH1*, *ATOH8*) genes expression of which in treated versus control RP was validated by ISH and by a reporter assay for *ATOH1* (Fig. 2K-P). This confirms and further extends our findings that RP, NC and dI1 interneurons fail to segregate in the absence of RA signaling (Rekler and Kalcheim, 2022).

**Fig. 3.**
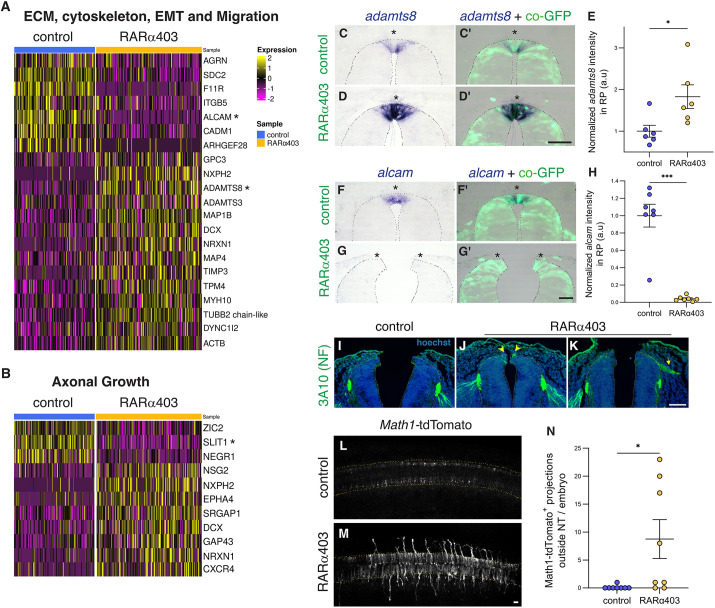
**Differential gene expression in the RP reveals changes in adhesive properties and axonal growth.** (A,B) Heatmaps of differentially expressed RP genes in response to RARα403. Selected genes (minimum linear fold change of ±1.3 and adjusted *P*-value <0.05) involved in (A) ECM, cytoskeleton, EMT, migration and (B) axonal growth and guidance. Genes validated *in vivo* are marked with an asterisk. (C-N) Validation on embryos electroporated with GFP along with control PCAGG or RARα403. RP is marked by asterisks. (C-E) ISH for *ADAMTS8*. *n*=6,6 for control and treated groups. (F-H) ISH for *ALCAM*. *n*=7,7 embryos. (I-K) Staining for neurofilament protein (NF) showing immunoreactive cells in treated RPs (arrowheads in J) and projections extending from the dorsal NT outward (arrow in K). *n*=9,9 embryos. (L-N) Embryos were electroporated as above, along with the Atoh1-tdTomato reporter. Atoh1-tdTomato^+^ axonal projections are confined to the control NTs yet abnormally extend from the dorsal NT into mesoderm (M). *n*=8 embryos for each treatment. *P<0.05, ***P<0.001, Mann–Whitney test. a.u., arbitrary units; NT, neural tube; RP, roof plate. See Table S7 for source data. Scale bars: 50 µm.

Inhibition of RA activity leads to an extended period of cell emigration from the dorsal NT subsequent to completion of the NC stage (Rekler and Kalcheim, 2022). Therefore, we expected a significant impact on genes involved in cytoskeletal function, EMT and migration. Indeed, the metalloproteases *ADAMTS8*, *ADAMTS15* and *TIMP3*, microtubules and myosin-associated genes such as *MAP4*, *TUBB2*, *TUBB3*, *MYH9* and -*10*, as well as genes involved in neuronal precursor migration such as *DCX*, were upregulated (Fig. 3A,C-E, Fig. S3C). Reciprocally, cell adhesion genes such as *ALCAM* were downregulated (Fig. 3A,F-H).

Lastly, a set of RP genes affected by RARα403 is related to axonal growth and guidance (Fig. 3B, Fig. S3B), for instance, *SLIT1* (Rekler and Kalchein, 2022), *CXCR4* (Kasemeier-Kulesa et al., 2010), the *EPHA4* and *EPHA3* receptors (Rodger et al., 2012), and *GAP43*. Consistent with this, neurofilament-expressing fibers were abnormally detected in the treated RP, indicating that RA is important for the correct patterning of neuronal cell processes. In addition, atypical neuronal projections extended out of the dorsal NT (Fig. 3I-K) and were shown, by a Atoh1 reporter, to correspond to dI1 interneurons (Fig. 3L-N) that normally project dorsoventrally within the NT (Yaginuma et al., 1994). These results extend our previous findings showing an infiltration of BarHL1-positive dI1 interneurons into the RP in the absence of RA signaling (Rekler and Kalcheim, 2022). The molecular mechanism accountable for the observed phenotypes among the aforementioned altered pathways is yet to be explored.

#### Single RP cells co-express RP, NC and dI1 traits

RP-derived RA signaling is crucial for the end of NC production, an effect that includes the downregulation of NC-specific genes such as *FoxD3*, *Snai2* and *Sox9* in the dorsal NT and consequent lack of NC EMT (Rekler and Kalcheim, 2022). Reciprocally, using a RP-specific *ALDH1A2* enhancer, we now show that upon late transfection of the above NC-specific genes, enhancer activity is reduced (Fig. S4). Thus, NC and RP genes stand in a mutually repressive interaction along a temporal axis, enabling a transition between the above states.

As shown in Fig. 2, lack of RA activity interfered with the above transition, as genes specific for RP, NC and dI1 were all evident in the RP cluster (see also Rekler and Kalcheim, 2022). It was therefore important to understand the effect of RA inhibition on the RP at single-cell resolution. To this end, control and treated RP cells were re-clustered into four sub-clusters, based on similarity in gene expression (Fig. S5, Table S2). Clusters 0 and 3 were primarily composed of control cells, showing few differences in gene expression. By contrast, clusters 1 and 2 consisted mainly of RARα403-treated cells, with cluster 2 demonstrating an intermediate expression profile partially similar to that of the control cells, perhaps owing to a lower transfection efficiency. In contrast, cluster 1, although retaining expression of the RP markers *RSPO1* and *RSPO3*, differed significantly from control clusters, encompassing the majority of the effects unveiled in the differential expression analysis (Fig. 2). Particularly, genes associated with both premigratory NC (*SNAI2*, *DLX5*, *SOX9*, *NOG*) and dI1 (*ATOH1*, *OLIG3*, *ATOH8*, *SOX9*) fates were highly expressed, hinting at a possible failure of fate segregation at the single-cell level.

**Fig. 4.**
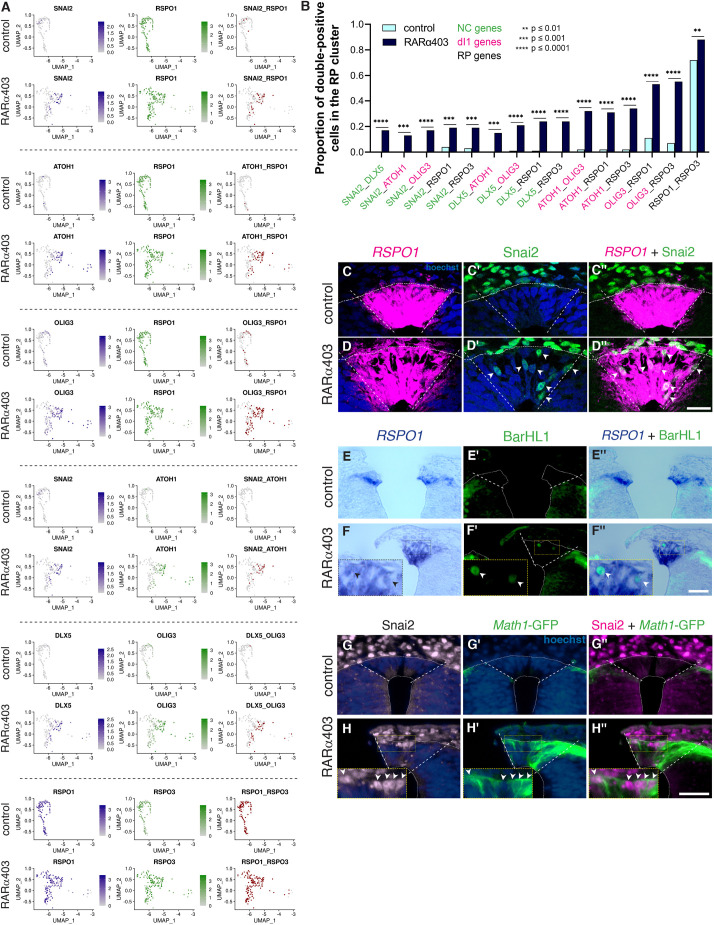
**Single RP cells co-express RP, NC and dI1 traits.** (A) Co-expression of NC, dI1 and RP genes in the RP cluster visualized on UMAPs of control and RARα403 samples. Double-positive cells (red) are significantly increased in treated samples compared with controls. Co-expression of *RSPO1* and *RSPO3* serves as positive control. (B) Co-expression of an array of gene pairs characterizing RP, NC and dI1 identities. **P<0.01, ***P<0.001, ****P<0.0001, Chi-square proportion test with a Benjamini-Hochberg FDR correction. (C-H″) Validations on embryos electroporated with control PCAGG or RARα403. (C-D″) Immunostaining for SNAI2 combined with fluorescent ISH for *RSPO1* shows the presence of SNAI2^+^*RSPO1*^+^ cells in the RP (arrowheads in D′,D″). *n*=8,8 for each treatment. (E-F″) Immunostaining for BarHL1 combined with ISH for *RSPO1* also reveals double-positive BarHL1^+^*RSPO1*^+^ cells in the RP (arrowheads in F-F″). *n*=6,6. (G-H″) Embryos were electroporated with a Atoh1-GFP reporter for dI1 labeling and immunostained for SNAI2. Note double-positive cells in the RP (arrowheads in H-H″). *n*=6,9 for controls and RARα403. Insets in F-F″ and H-H″ show higher magnifications of the respective boxed areas. Dashed lines mark the RP in C-H″. dI1, dorsal interneurons 1; NC, neural crest; RP, roof plate. Scale bars: 50 µm.

Therefore, we examined whether RP, NC and dI1 genes are co-expressed in individual RP cells of treated embryos. To this end, cells with a minimum of two reads per gene were considered positive. Fig. 4 demonstrates that, whereas control RP clusters contained few or no double-positive cells, a significant proportion of the treated RP cells co-expressed the RP marker *RSPO1* together with either NC (e.g. *SNAI2*) or dI1 (e.g. *ATOH1* or *OLIG3*) markers. Remarkably, co-expression of NC together with dI1 markers was detected as well (e.g. *SNAI2*-*ATOH1* and *DLX5*-*OLIG3*). Co-expression of *RSPO1* and *RSPO3* in control and in treated RP cells served as positive controls. These findings, in conjunction with additional gene pairs tested, are summarized in Fig. 4B and further validated by combined expression of *RSPO1*, SNAI2 and BarHL1, and a reporter assay for *ATOH1* (Fig. 4C-H″). Collectively, these results suggest that, at some time during development, individual dorsal NT cells have at least three potential fates: NC, RP and dI1. Here, we show that normal segregation between these fates, in both time and space, is regulated by RA.

#### Segregation of RP from dI1 interneurons requires RA inhibition of Notch signaling

**Fig. 5.**
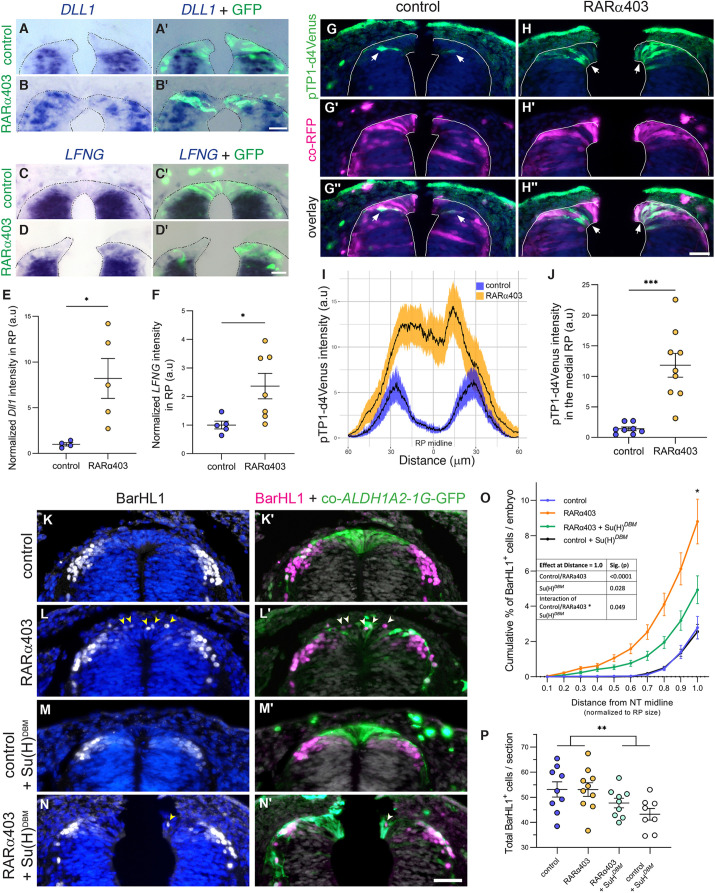
**Segregation of RP cells from dI1 interneurons requires inhibition of Notch signaling by RA.** (A-J) Embryos were electroporated with control PCAGG or RARα403 at E2.5 and analyzed at E4. (A-F) ISH of *DLL1* (A-B′,E) and *LFNG* (C-D′,F), showing positive gene expression ventral to RP in controls, expanding into the RP in treated embryos. *n*=4,5 (*DLL1*) and 5,7 (*LFNG*) for control and RARα403 groups. (G-J) Electroporated embryos with RFP and the pTP1-d4Venus to monitor Notch activity. Arrows in G-H″ mark pTP1 signal, showing a notable expansion into the RP domain of treated embryos and no signal in control RP. (I) Quantification of signal distribution in RP and adjacent interneurons. (J) Quantification of pTP1 signal intensity in the medial 30 µm of the dorsal NT. (K-P) Embryos were electroporated as above, with addition of either a control vector or Su(H)*^DBM^* under a RP-specific *aldh1a2* 1G enhancer. GFP driven by the same enhancer served as control. Arrowheads in L,L′,N,N′ mark BarHL1^+^ cells inside the RP. Note the reduction of BarHL1^+^ cells in the RP between RARα403 alone (L) and RARα403 combined with Su(H)*^DBM^* (N). (O) Quantification of the percentage distribution of individual BarHL1^+^ cells located inside the RP as a function of distance from the dorsal midline, normalized to RP size. Two-way ANOVA was performed on the data at Distance=1.0, showing a significant effect of Su(H)*^DBM^* in reducing the phenotype of RARα403, thus resulting in fewer BarHL1^+^ cells inside the RP. (P) Quantification of the total number of BarHL1^+^ cells. *n*=9,10,9,8 embryos for control, RARα403, RARα403+Su(H)*^DBM^* and control+Su(H)*^DBM^* groups, respectively. *P<0.05, **P<0.01, ***P<0.001, Welch's *t*-test for A-J, two-way ANOVA test for O,P. a.u., arbitrary units; NT, neural tube; RP, roof plate. See Table S8 for source data. Scale bars: 50 µm.

Whereas the temporal separation between NC and RP traits depends on repression of BMP/Wnt activities by RA (Rekler and Kalcheim, 2022), the spatial segregation between RP and adjacent dI1 interneurons is less well understood. We demonstrated that Notch signaling is responsible for the formation of both RP and dI1 interneurons, likely by defining a boundary between both cell types (Ofek et al., 2021). Reciprocally, in the absence of RA activity, dI1 neurons infiltrate into the RP domain, suggesting that the boundary between them is compromised (Rekler and Kalcheim, 2022). Examination of the differentially expressed genes in response to RARα403 in the RP, revealed an upregulation of Notch-related genes (Fig. 2A), implying a potential repression of the Notch pathway by RA. Next, ISH of two central Notch pathway components, *DLL1* and *LFNG*, confirmed that their expression broadened into the RP domain of the RARα403-treated embryos. This stands in contrast to controls, in which the dorsal limit of gene expression corresponded to the boundary between RP and dI1 neurons (Fig. 5A-F; Ofek et al., 2021).

Subsequently, we visualized Notch activity in the absence of RA signaling, employing the pTP1 unstable reporter. Fig. 5G-J shows that control embryos displayed a narrow domain of Notch activity at the RP-dI1 boundary, whereas inhibition of RA activity led to its expansion throughout the entire RP. Together, these results indicate that RA represses Notch activity in the RP, confining it to boundary regions at this stage.

Thus, we reasoned that suppression of Notch signaling in cells deprived of RA activity would rescue the boundary between RP and BarHL1-positive dI1 interneurons. For this purpose, we locally inhibited the Notch pathway in the dorsal NT by electroporating Su(H) harboring a mutation in the DNA-binding domain [Su(H)*^DBM^*], expressed under the regulation of an RP-specific *ALDH1A2* enhancer. Using this construct, combined with inhibition of RA activity, we reveal that although RA inhibition alone resulted in the abnormal presence of multiple BarHL1^+^ cells in the treated RP, this effect was significantly diminished when Notch activity was simultaneously inhibited (Fig. 5K-O). As anticipated, Su(H)*^DBM^* alone exhibited no discernible effect on dI1 cell distribution compared with control embryos, as Notch activity is typically absent in the RP itself (Fig. 5O). A mild decrease in the overall count of BarHL1^+^ cells was monitored in Su(H)*^DBM^*-treated embryos in both control and RARα403 conditions (Fig. 5P), consistent with the requirement of Notch activity for the generation of dI1 interneurons. Yet the main effect differentiating between controls with or without Su(H)*^DBM^* and RARα403-treated embryos with or without Su(H)*^DBM^* was the spatial distribution of interneurons.

Together, these results suggest that by repressing Notch activity in the RP and restricting it to the RP-dI1 interphase, RA indirectly defines the boundary between RP and dI1 domains.

### RA signaling induces fate segregation of late emerging NC-derived cell types

#### Gene expression profiles in control and RARα403-treated NC derivatives

The last NCs to exit the trunk-level NT generate sequentially neural progenitors that settle in the periphery of DRG, and melanocytes (Krispin et al., 2010b; Loring and Erickson, 1987). Because of the technique utilized to isolate NTs, certain NC-derived cells expressing Msx1-Citrine and located in the mesenchyme near the NT were incorporated into our scRNA-seq analysis as clusters 11, 19 and 20 (Fig. 1D,G). Marker gene expression in the control sample revealed three distinct populations within the peripheral clusters: melanocytes (cluster 20), DRG progenitors (pDRG; cluster 11) and differentiating DRG neurons (cluster 19) (Fig. S6A-C). Apart from cell type-specific genes, pDRG uniquely expressed *EDNRB*, whereas melanocytes expressed *EDNRB2*, two guidance receptors responsible for ventral versus dorsolateral routes of NC migration, respectively (Fig. S6D) (Harris et al., 2008; Nataf et al., 1996). The differentiating DRG cluster was characterized by expression of specific sensory neuron genes such as *NEUROD1*, *ISL1* and *BRN3*, as well as by generic neuronal genes such as *DCX*, *NEFL* and *TAGLN3* (Fig. S6A-C)*.*

By comparing RARα403-treated and control samples, several gene categories displayed significant changes (Fig. S7). Notably, these effects were prominent in the pDRG and melanocyte clusters, whereas minimal differential gene expression was observed in differentiating DRG neurons. Importantly, a significant upregulation of glial genes such as *MBP*, *PMP22*, *KNDC1*, and of *VWDE/SERAF* (Suzuki et al., 2015; Wakamatsu et al., 2004), was detected not only in the treated pDRG cluster, but also in the melanocyte group. In addition, *SOX2* was upregulated in pDRG, suggesting the maintenance of a progenitor state.

An increase in the expression of ECM-related genes (such as *COL2A1*, *LAMB1*, *LAMA4*) and genes associated with adhesion, EMT and migration (such as *DUSP7*, *OLFM1*, *MEF2C*, *CDH19*) was also observed in the treated pDRG and melanocyte clusters (Fig. S7), suggesting that absence of RA activity encourages cellular remodeling.

#### A bridge connecting pDRG with melanocytes corresponds to the late-emigrating NCs observed in RARα403-treated embryos

**Fig. 6.**
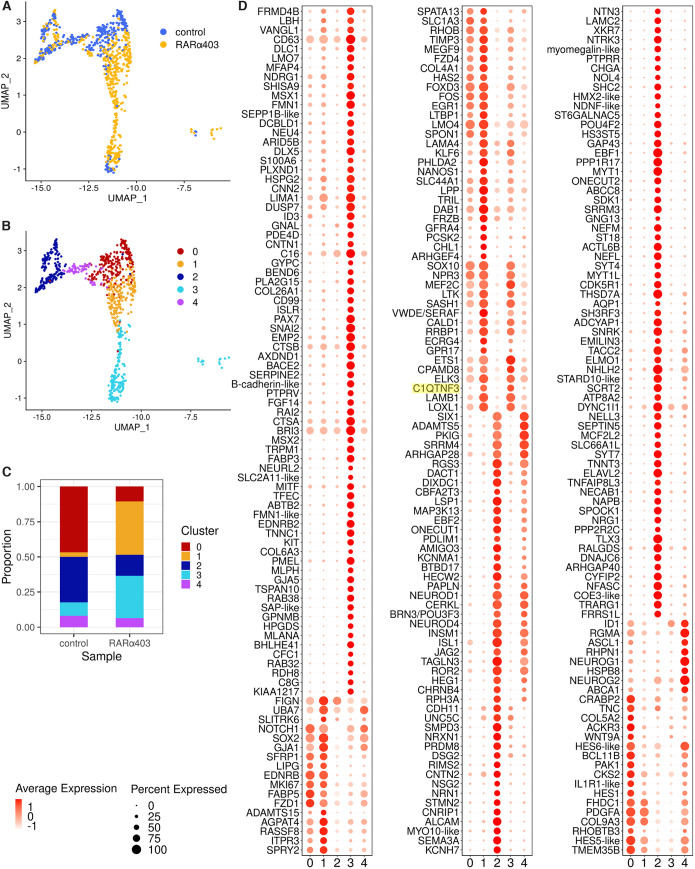
**Re-clustering of NC derivatives reveals a bridge connecting DRG and melanocyte progenitors.** (A) UMAP of the peripheral clusters (11, pDRG; 19, differentiating DRG neurons; 20, melanocytes) colored by sample. See Fig. 1D,F for cluster identification. (B) Re-clustering generated five subclusters (0-4). Cluster 1 bridges between pDRG and melanocytes. (C) Proportions of the different subclusters, compared between control and RARα403 samples. Cluster 1 is predominant in the treated sample. (D) Dot plots illustrating gene expression in the five subclusters. Marker analysis was conducted on the data from peripheral clusters only. Highlighted in yellow is *C1QTNF3*.

**Fig. 7.**
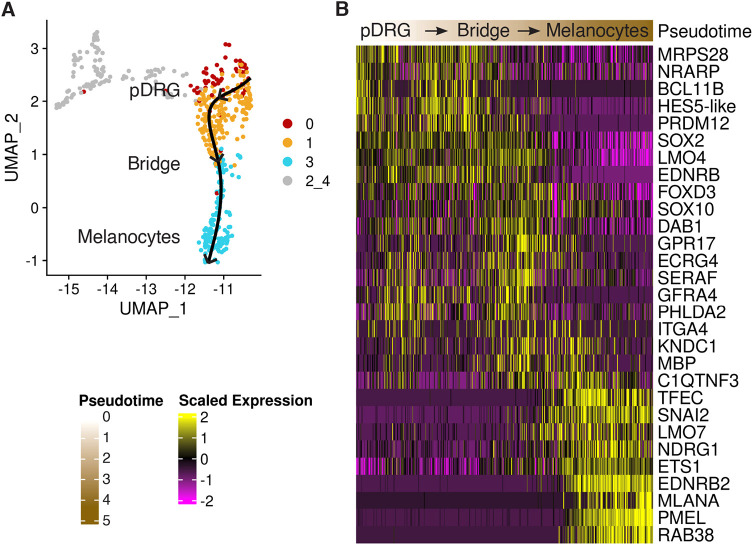
**Progressing expression of selected genes along the pseudotime in peripheral clusters.** (A) UMAP plot of pDRG-bridge-melanocyte cells. Each circle is a cell. Cells are colored according to the clusters (re-clustering of DRG-melanocytes). Clusters 2 and 4 containing neuroblasts and neurons are in gray. Trajectory inference with cluster 0 set as the starting cluster and cluster 3 set as the end cluster. Eleven cells distant in the UMAP dimensionality reduction were excluded. The fitted principal curve is shown in black. (B) Expression profiles of selected genes in pDRG, bridge and melanocyte clusters (0, 1 and 3, respectively) of RARα403-treated cells. Cells are ordered by the pseudotime, shown as top annotation.

Whereas pDRG and melanocyte clusters were separated in the control UMAP, a ‘bridge’ connecting these clusters emerged in the RARα403 sample (Fig. 6A). To characterize this, the three peripheral groups were re-clustered, resulting in five distinct subpopulations (Fig. 6B,C, Table S3) with unique gene expression profiles (Figs 6D and 7, Fig. S8). The bridge was primarily identified as cluster 1 but also contained part of cluster 3. Cluster 1 was prominent in the treated sample but nearly absent in control (Fig. 6A-C). Marker examination unveiled that cluster 1 shows a range of glia-related genes, including *EDNRB*, *FOXD3*, *SERAF*, *SOX2*, *GPR17*. Some markers of cluster 1 were also shared with cluster 3 (originally the melanocytic cluster). These included *SOX10*, *SASH1*, *CALD1*, *NPR3*, *MEF2C* and *LOXL1*. Other noteworthy genes shared between clusters 1 and 3 were *DLC1*, which mediates cranial NC delamination (Zhao et al., 2024), and *CCN2*, a glial ECM protein with EMT properties (Zhou et al., 2023). Thus, the emergence of the bridge cluster in the treated sample confirms the maintenance of a link between pDRG and melanocytes in absence of RA activity.

**Fig. 8.**
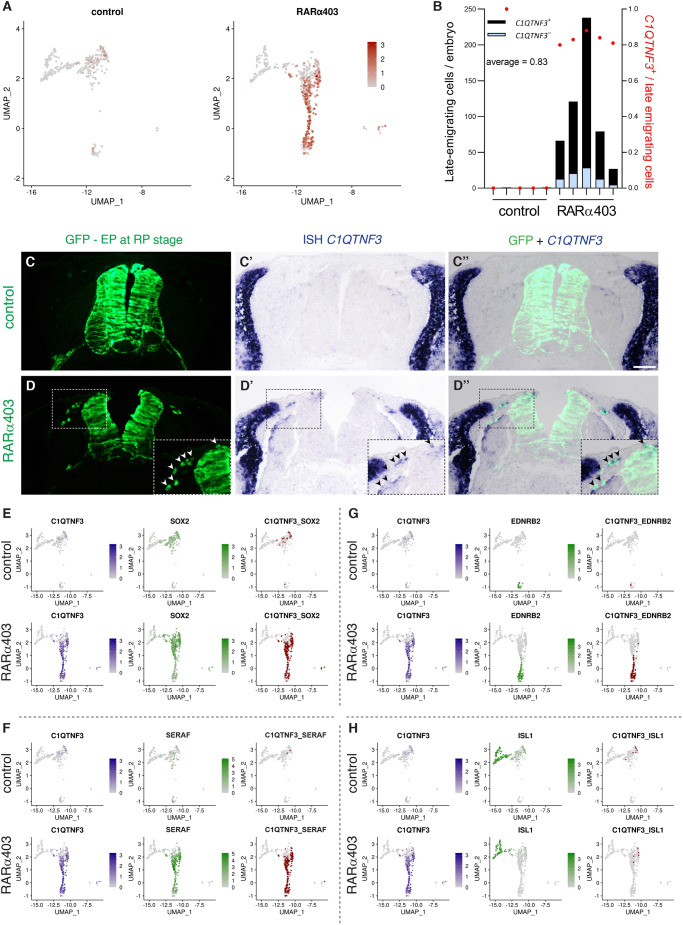
**The bridge connecting DRG and melanocytes corresponds to late-emigrating cells and is defined by *C1QTNF3* expression.** (A) UMAPs depicting *C1QTNF3* expression in the peripheral clusters of control and RARα403 samples. Expression is primarily restricted to cells in the treated sample that connect the pDRG and melanocytes. (B-D″) *C1QTNF3* in late-emigrating cells. Embryos were electroporated at E2.5 with either control PCAGG or RARα403, and NTs were subjected to a second electroporation with a GFP plasmid at E3.5 to label late NC cells. GFP^+^ late-emigrating cells at E4 were subjected to *in situ* hybridization for *C1QTNF3.* (B) Quantification of the number of late-emigrating cells per embryo with and without expression of *C1QTNF3*. (C-D″) Whereas virtually no positive cells exited the control NTs, numerous NC cells left the NT by E4 in the treated samples. In D-D″, insets (magnifications of the respective boxed areas) and arrowheads show the presence of emigrated cells co-expressing GFP and *C1QTNF3*. *n*=5,5 for each treatment. (E-H) Co-expression of *C1QTNF3* with other genes in both control and RARα403 samples visualized on UMAPs of the peripheral clusters. Double-positive cells are marked in red. Negligible co-expression was observed in the control sample, significant co-expression of C1QTNF3 with progenitor (*SOX2*), glia (*SERAF*) and melanocyte (*EDNRB2*) genes in the treated sample, but no co-expression with the neuronal gene *ISL1*. See Fig. S9 for additional visualizations, and Fig. 11A for quantification. See Table S9 for source data. Scale bar: 50 µm.

We previously reported that inhibition of RA signaling in the dorsal NT prior to NC-to-RP transition extends the period of NC production and emigration into the RP stage (E4), when NCs are no longer produced (Rekler and Kalcheim, 2022). Given that these late-emigrating cells were unique to RARα403-electroporated embryos, we set out to examine whether bridge cells, also specific to the treated embryos, correspond to the late-emigrating cells observed *in vivo*. The most distinctive marker of the bridge cells was the adipokine *C1QTNF3* (Li et al., 2017), a factor with unknown function in the present context. *C1QTNF3* was highly specific to bridge cells and absent from additional cell subsets (Fig. 6D). ISH confirmed *C1QTNF3* expression in 83% of late-emigrating cells at E4, but not in neural cells of controls (Fig. 8A-D″). Furthermore, co-expression analysis of *C1QTNF3* with an array of genes revealed that a significant proportion of bridge cells co-expressed *SOX2*, further confirming their persistent progenitor state. Additionally, they co-expressed an assortment of glial (e.g. *SERAF*, *MBP*, *KNDC1*, *EDNRB*) and melanocytic (e.g. *EDNRB2*, *PMEL*, *TFEC*) genes, but not neuronal genes (e.g. *ISL1*, *NEUROD1*, *NTRK3*) (Fig. 8E-H, Fig. S9).

**Fig. 9.**
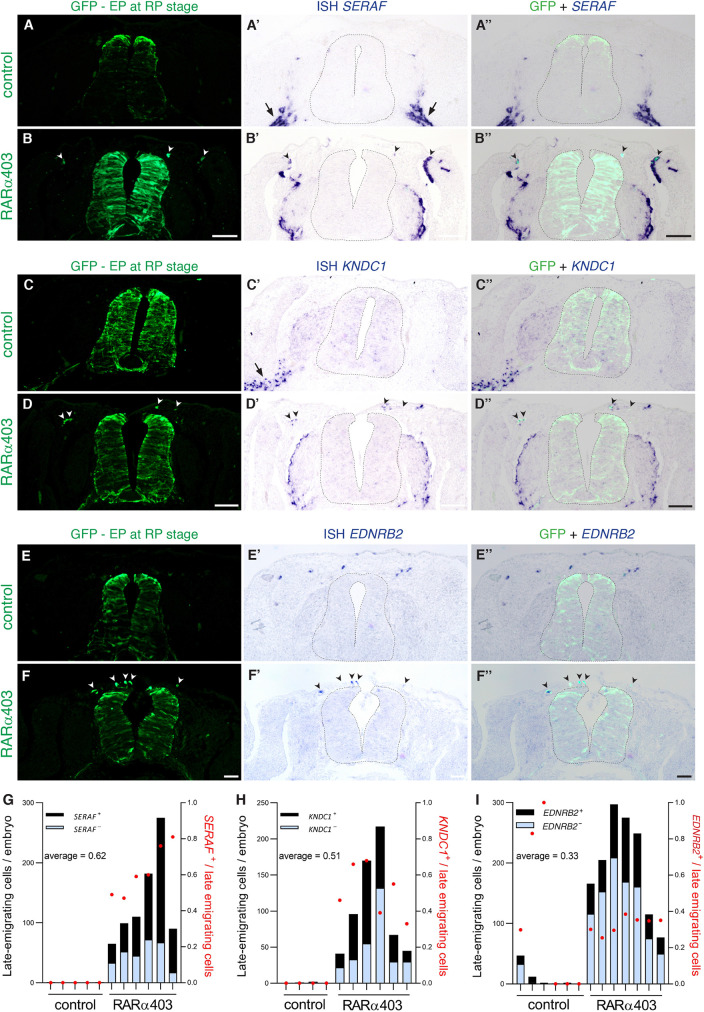
**The late-emigrating bridge cells express glial and melanocyte markers.** (A-I) *In vivo* validation of the expression of *SERAF* and *KNDC1* (A-D″,G,H) and *EDNRB2* (E-F″,I) in late-emigrating cells. Embryos were electroporated at E2.5 with either control PCAGG or RARα403, and NTs were labeled with GFP via a second electroporation at E3.5. GFP^+^ late-emigrating cells at E4 co-expressing the various genes were counted. (A-D″) ISH for *SERAF* (A-B″) and *KNDC1* (C-D″). Arrows indicate normal expression in progenitors residing along nerves, and arrowheads mark GFP^+^ late-emigrating cells in DRG and throughout the mesoderm restricted to treated samples (see Fig. 10 for additional images). (E-F″) ISH for *EDNRB2*. Note expression in control melanocytes (E,E′), and co-expression with GFP in late-emigrating cells (F-F″). Scale bars: 50 µm. (G-I) Quantification in late-emigrating cells. *n*=5,6 for *SERAF*; *n*=4,6 for *KNDC1*; *n*=6,7 for *EDNRB2*, in control and RARα403, respectively. See Table S9 for source data.

**Fig. 10.**
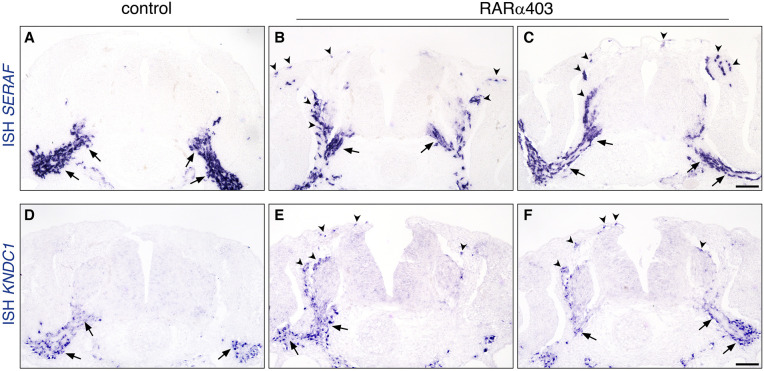
**Glial markers are ectopically expressed upon inhibition of RA activity.** (A-F) Embryos were electroporated with control PCAGG or RARα403 at E2.5, fixed at E4 and subjected to *in situ* hybridization for expression of *SERAF* (A-C) or *KNDC1* (D-F). Arrows indicate normal expression sites along nerves in both control and treated embryos, whereas arrowheads highlight positive cells in the mesoderm including sites of melanocyte migration through the dermis, and DRG periphery, seen exclusively in the treated embryos (B,C,E,F). Scale bars: 50 µm.

Moreover, ISH at E4 for the glia-specific genes *SERAF* and *KNDC1* showed expression in Schwann cells residing along the spinal nerve of control embryos (Figs 9A-A″,C-C″, 10A,D, arrows). In treated embryos, they were additionally apparent in the DRG periphery, in the mesenchyme surrounding the NT and also in dermis, where melanocytes typically migrate (Figs 9B-B″,D-D″, 10B,C,E,F, arrowheads). Specifically, *SERAF* and *KNDC1* were expressed by 62% and 51% of late-emigrating cells, respectively (Fig. 9G,H). In addition, EDNRB2, transcribed by melanocytes of control embryos (Fig. 9E-E″), was also detected in 33% of late-emigrating cells and was mainly detected throughout the dermis (Fig. 9F-F″,I). A heatmap depicting the trajectory of the precedent and of additional genes arranged by pseudotime further illustrates their dynamical behaviors (Fig. 7; see also Kastriti et al., 2022 for PRDM12 and ITGA4).

To examine bridge cell temporal dynamics, ISH for *C1QTNF3*, *SERAF* and *EDNRB2* was performed at E5 and E6. At E5, control samples showed patterns similar to E4 for all genes. In RARα403 samples, few *C1QTNF3*^+^ cells remained adjacent to the dorsal NT (Fig. S10A-D), many *SERAF*^+^ cells were scattered in the dorsal dermis and epidermis in addition to their normal distribution along nerves (Fig. S11A-D), and *EDNRB2*^+^ melanocytes were present in both dermis and epidermis but additionally were also localized in the mesenchyme near the NT (Fig. S12B,D, arrowheads), and along nerve fibers likely extending abnormally from dorsal interneurons outside the confines of the NT (Fig. S12, arrowheads in C; see also Fig. 3L,M). By E6, no differences were observed between control and treated conditions (Figs S10,S11,S12), suggesting that bridge progenitors with both glia and melanocyte properties persist for at least 2 days after normal NC production ends.

Collectively, these findings confirm that late-emigrating cells express glia and melanocytic genes, providing the *in vivo* equivalent of the bridge cells connecting pDRG and melanocytic clusters in the scRNA-seq.

#### A common glia-melanocyte (GM) progenitor is revealed in the absence of RA signaling

**Fig. 11.**
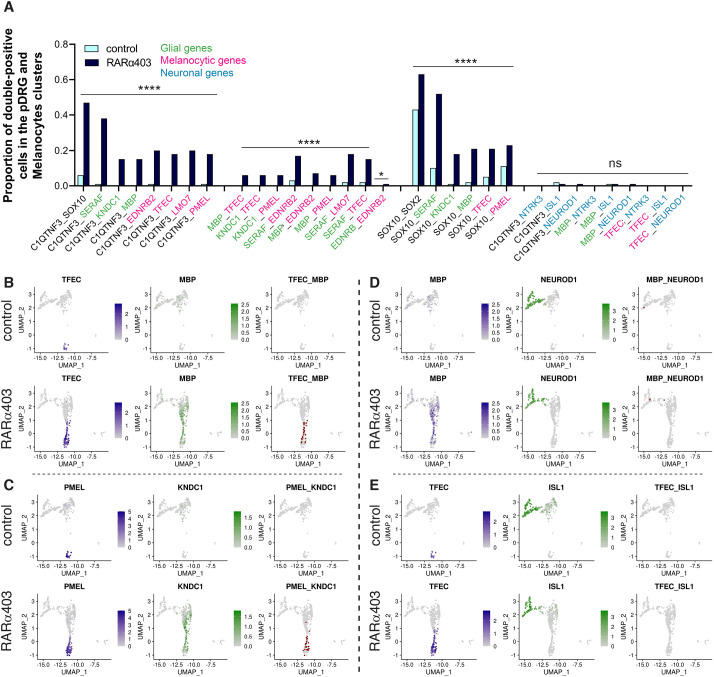
**Single cells in the bridge co-express glia and melanocyte genes.** (A) Testing for the co-expression of an array of gene pairs in pDRG and melanocyte clusters (see Materials and Methods). From left to right, *C1QTNF3* combined with glia or melanocyte genes, glia combined with melanocyte genes, *SOX10* combined with glia and melanocyte genes, and neuronal genes with *C1QTNF3* and glia/melanocyte markers. (B-E) Visualization of selected pairs from the above quantifications on UMAPs of the peripheral clusters, separated into control and RARα403 samples. ****P<0.0001, Chi-square proportion test with a Benjamini-Hochberg FDR correction. ns, not significant.

The results described above suggest that bridge cells might represent a transitional state preceding commitment to either glial or melanocytic fates. This would predict that individual glial progenitors co-express melanocyte traits. In fact, the existence of a bipotent NC-derived GM progenitor has been proposed based on *in vitro* studies (Dupin et al., 2000). Moreover, Schwann cell progenitors have also been demonstrated to generate melanocytes *in vivo* (Adameyko and Lallemend, 2010; Adameyko et al., 2009; Nitzan et al., 2013). Thus, we examined co-expression of glia, melanocyte and neuronal genes in single cells within the pDRG and melanocyte clusters. Co-expression of glia with melanocyte genes was evident at E4 in a subset of the RARα403-treated sample (Fig. 11A-C, Fig. S13A-E), but were nearly absent in controls. In contrast, neuronal genes were restricted to the differentiating DRG cluster, showing no co-expression with either glial or melanocyte genes (Fig. 11A-E, Fig. S13F). *SOX10* is normally transcribed in both early glia and pigment lineages (Cheng et al., 2000; Saldana-Caboverde et al., 2015). Consistent with this, it was present in almost all cells within the pDRG and melanocyte clusters of control samples, serving as a positive control in the co-expression analysis. Furthermore, an enhanced proportion of *SOX10*^+^ cells combined with either glia or melanocyte genes was detected upon treatment (Fig. 11A). Hence, lack of RA signaling supports the maintenance of a common GM progenitor and prevents its timely segregation into individual fates.

To obtain a dynamical view of the above findings, we estimated RNA velocities of the treated sample using the dynamical model implemented in scVelo (Bergen et al., 2020). For improved visualization, a principal component analysis (PCA) projection instead of a UMAP was applied (Fig. S14A,B). Cell cycle analysis suggested that the separation into clusters is unlikely to be affected by the cell cycle phase (Fig. S14C). In our dataset, RNA velocity revealed a clear bifurcation stemming from cluster 1, the bridge, leading either towards melanocytes (cluster 3) or towards pDRG (cluster 0) followed by neuronal progenies (clusters 4 and 2) (Fig. S14D). Next, we interrogated the behavior of single genes in each cluster. For example, cluster 0 (pDRG) top velocity genes such as *CNKSR3* and *CADM1*, stabilized towards either melanocytes or neurons, respectively (Fig. S14E). Cluster 1 genes with highest velocity in bridge cells, streamed towards pDRG and melanocytes (Figs S14F, S15A). Furthermore, cluster 3 genes streamed towards a melanocyte fate yet were also upregulated in bridge cells (Figs S14H, S15C). Thus, the dynamic trajectories leading from bridge progenitors towards segregated fates are effectively illustrated by analyzing single genes. In contrast, genes with top velocity in clusters 2 or 4 were mainly expressed in differentiated or differentiating neurons, respectively, consistent with their patterns of mRNA expression (Figs S14G,I, S15B,D). Finally, scVelo latent time analysis further confirmed that pDRG and bridge cells are produced earlier than melanocytes and neuronal cells (Fig. S14J).

## DISCUSSION

By implementing a combination of *in ovo* gene misexpression and single-cell RNA profiling, we demonstrate that RA is an essential component of the nascent RP organizer, which plays a key role in segregation of cell fates in both time and space.

We recently reported that RA is responsible for the end of NC production and emigration, hence segregating between PNS (NC) and dorsal CNS (RP) fates. This is accounted for by repressing BMP and Wnt activities (Rekler and Kalcheim, 2022), both found to be necessary for the onset of NC EMT (Burstyn-Cohen et al., 2004; Sela-Donenfeld and Kalcheim, 1999). The present scRNA-seq analysis reveals the RA-dependent upregulation of specific BMP inhibitors in the RP, previously uncovered in a differential transcriptome between premigratory NCs and RP cells (Ofek et al., 2021; Rekler and Kalcheim, 2022). These repressors are likely to mediate, at least partially, the loss of responsiveness to BMP, as documented for early misexpression of Hes1 (Nitzan et al., 2016).

In the absence of local RA activity, not only NCs fail to separate from RP cells in the temporal dimension, as we also observe an invasion of the RP by dI1 interneurons, but not by more ventral interneuron types. This suggests that, even if an RP forms without RA, it fails to spatially separate from its ventrally localized neighbors. Notably, Notch signaling is both necessary and sufficient for the formation of a RP and of dI1 interneurons (Ofek et al., 2021), and here we further uncover that separation of RP cells from dI1 neurons is elicited by RA via restriction of Notch signaling to the boundary region between both cell types. This is consistent with loss of the constitutive expression of the boundary gene Hes1/4 (Baek et al., 2006; Osoŕio et al., 2014; Osório et al., 2019) in the treated RP, and may also be related to the observed downregulation of *ISM1*, a nodal repressor expressed in the midbrain–hindbrain boundary (Osoŕio et al., 2014, 2019). Associated with the compromised separation of the RP from dI1 lineages, absence of RA input might lower the mechanical tension required for keeping the boundary straight and precluding cell intermixing (Cheng et al., 2004).

Our results further uncovered changes in various axonal guidance genes. Consistently, the growth of dI1 axons was aberrant and extended outside the confines of the NT. It remains to be clarified which of the pathways modified by lack of RA (*SLIT1*, *CXCR4*, *EPHA4*, etc.) accounts for the phenotype observed. Together, our results illustrate that RA is used reiteratively and contextually in combination with BMP, Wnt and Notch, providing further insight into how a small repertoire of signaling pathways interacts to ensure that correct cell types are specified at the right time and place. In addition, despite being consistently produced in the paraxial mesoderm (Bernheim and Meilhac, 2020), the RA responsible for the regulatory functions here defined originates from a novel source: the nascent RP (Rekler and Kalcheim, 2022). This emphasizes the concept that different sources of the factor are associated with distinct functions (Del Corral et al., 2003; Diez del Corral and Morales, 2014; Martínez-Morales et al., 2011).

Along this line, loss of RP-derived RA activity is associated with substantial cellular alterations. Treated RPs are loosely organized, as evidenced by loss of cell adhesion molecules, upregulation of metalloproteases, maintenance of a discontinuous basal lamina and upregulation of EMT factors, compared with the normal RP, which is an epithelial structure with distinct boundaries (this study and see also Nitzan et al., 2016). A highlight of this plasticity is an altered state of cellular commitment, as the RP of RARα403-treated embryos remains proliferative (Rekler and Kalcheim, 2022), and exhibits the presence of single progenitors co-expressing NC, RP and dI1 markers. Whereas the lack of segregation between NC and RP lineages is less surprising owing to their shared derivation from a FOXD3^+^ lineage, the presence of a common progenitor for NC/RP and dI1 cells is more intriguing and suggests that boundaries between domains serve not only structural purposes but also facilitate the correct segregation into distinct cell types. For instance, in controls dI1 progenitors exhibit high BMP activity compared with their RP neighbors. Perhaps the extension of BMP activity to the RP of RARα403-treated embryos (Rekler and Kalcheim, 2022), coupled with aberrant Notch activity, enables dI1 features to be expressed inside the RP domain and prevents phenotypic segregation.

Mechanical harvesting of the NTs for RNA-seq purposes also enabled the collection of Citrine^+^ NC-derived cells encountered in the mesoderm adjacent to the NT. These cells were also likely to be exposed to dorsal NT-derived RA, as its synthesis begins at the late NC stage in dorsal NT, just prior to the establishment of the definitive RP (Ofek et al., 2021). Bioinformatic analysis revealed that, in addition to distinct clusters composed of pDRG, DRG neurons or melanocytes, all apparent under control conditions, an additional cluster appeared only in absence of RA activity. We termed this cell group a ‘bridge’ as it connects pDRG with melanocytes. Molecularly, bridge cells are composed of both pDRG with a glial character and/or also melanoblasts, but not neuroblasts or neurons. Most notably, GM traits were expressed by single cells of treated samples, yet few or no cells co-expressed these genes under control conditions. Hence, bridge cells represent a ‘hybrid’ state along the hierarchy of cell specification, unravelling *in vivo* the presence of a bi-fated intermediate cell type with both GM progenitor properties, and further substantiating the notion that RA is necessary for fate segregation between the above peripheral lineages.

Our findings are in line with previous studies highlighting the plasticity of the melanocyte-glial state *in vitro* by showing that these phenotypes can interconvert under specific medium conditions (Ciment, 1990; Dupin et al., 2000; Real et al., 2006). In mice, the decision to become glia or melanocytes was shown to be driven by Wnt signaling that acts upstream of Sox10 and Pax3 (Colombo et al., 2022), or by NR2F1 in a model of Waardenburg syndrome (Bonnamour et al., 2022). Moreover, mice carrying spontaneous or targeted mutations of *Sox10* lack satellite glia and Schwann cells and exhibit pigmentation defects, but neurogenesis is initially unaffected (Britsch et al., 2001). Furthermore, melanoma progression from normal pigment cells is accompanied by a de-differentiation state in which melanocytic markers such as *DCT* and *MITF* are downregulated and glial/melanoblast markers are instead upregulated (Centeno et al., 2023).

Most notably, Schwann cell progenitors, a specialized glial subset, generate either Schwann cells or hypaxial and limb melanocytes depending on the level of contact with peripheral nerves (Adameyko et al., 2009). This process is regulated by cross-repressive interactions between FOXD3 and MITF (Kos et al., 2001; Nitzan et al., 2013; Thomas and Erickson, 2009). Furthermore, at cranial levels of the mouse embryonic axis, cross-regulatory interactions between Sox2 and Mitf were shown to consolidate a Schwann cell progenitor versus melanocyte fate, respectively (Adameyko et al., 2012). In addition to melanocytes derived from Schwann cell progenitors, epaxial pigment cells have a direct origin from the NC following migration through a dorsolateral pathway (Nitzan et al., 2013). Our RNA-seq analysis supports the presence of two distinct pigment cell populations: one with melanocytic genes being exclusively expressed in the melanocyte cluster and another shared with bridge and presumptive glia cells. This raises the interesting hypothesis that bridge cells might represent a population of progenitors able to generate ganglionic glia, Schwann cells or melanocytes. Along this line, in the absence of RA signaling, Schwann cell progenitors might become biased towards a melanocyte lineage and generate melanocytes for a longer time past the normal period of NC production. This is consistent with the maintenance in bridge cells of *FOXD3*, *SOX2* and *SOX10*, markers of the precursor state. RNA velocity and latent time analyses support the notion that bridge cells are early progenitors from which a bifurcation into either melanocytes or neural progenitors evolves.

In the current study, we have also delineated the bridge subpopulation as corresponding to late-emigrating NC progenitors that become apparent only in the absence of RA signaling. A noteworthy finding is that over 80% of these late-emigrating cells express a distinctive gene, which is novel in the present context: the adipokine C1QTNF3 (CTRP3). C1QTNF3, known as an anti-inflammatory cytokine (Chen et al., 2021; Li et al., 2017; Lv et al., 2022), plays a role in stimulating macrophage chemotaxis (Micallef et al., 2022). Intriguingly, it is one of the 12 prognostic genes linked to recurrence-free survival in human cutaneous melanoma (Karapetyan et al., 2022) and is associated with pigmentary traits (Farré et al., 2023). Our analysis revealed that late-emigrating, *C1QTNF3*^+^ bridge cells co-express both glial and melanocyte genes, excluding neuronal genes. This suggests a potential involvement in the development of glia–melanocyte interactions, warranting further exploration into potential developmental functions.

Another intriguing gene in this context is *SERAF*. *SERAF* was initially identified in avian embryos as an early, Schwann cell-specific gene regulated by SOX10 (Suzuki et al., 2015; Wakamatsu et al., 2004). In control embryos, *SERAF* was expressed in Schwann cell progenitors along peripheral nerves. However, in treated embryos, it marked over 60% of late-emigrating cells and was distributed throughout the mesenchyme, including subectodermal sites and the periphery of DRG, a region where satellite glia develop. This suggests that endogenous RA signaling may restrict *SERAF* expression to the Schwann cell lineage, and the absence of RA allows for the emergence of a multipotent progenitor that includes satellite glia. In contrast, bridge cells are unlikely to represent boundary cap cells (Hjerling-Leffler et al., 2005; Maro et al., 2004), as no *KROX20* (*EGR2*) was detected, and the expression of *PRSS56* (Coulpier et al., 2010) remained unchanged in the absence of RA activity.

Collectively, our data not only shed light on the lineage potential of RA-deficient cells but may also reveal aspects of normal fate segregation influenced by endogenous RA activity. In this context, it is tempting to suggest the existence of a common NC/RP/dI1 progenitor at some point in early neural tube development. Moreover, our findings hint at the possibility that at least a subset of glia and melanocytes evolves from a shared GM progenitor during normal development.

## MATERIALS AND METHODS

### Experimental model

Fertilized quail (*Coturnix coturnix japonica*) eggs were obtained from commercial sources (Moshav Mata), kept at 15°C and then incubated at 38°C to the desired stages. All experiments were performed on embryos younger than E5 and were therefore not subject to IACUC regulations.

### Expression vectors and *in ovo* electroporation

The following expression vectors were used: control pCAGG, pCAGGS-eGFP (Krispin et al., 2010b), pCAGGS-RFP (Ofek et al., 2021), pCAGGS-RARα403 (Rekler and Kalcheim, 2022), Msx1(ehn-264)-Citrine (Williams et al., 2019), Atoh1-tdTomato-F, Atoh1-GFP (Dumoulin et al., 2021), aldh1a2-1G-eGFP (Castillo et al., 2010), aldh1a2-1G-RFP (subcloned from Castillo et al., 2010; eGFP replaced with RFP), aldh1a2-1G-xSu(H)1*^DBM^* [subcloned from Castillo et al., 2010; replaced with xSu(H)1*^DBM^* (Jen et al., 1997)], pCA-FoxD3-IRES-EGFP (Dottori et al., 2001), pCAGGS-cSnai2(Slug)-IRES-nls-GFP, pCAGGS-Sox9-IRES-nls-GFP (Cheung et al., 2005) and pTP1-d4Venus (Matsuda et al., 2005).

For NT electroporation, DNA (4 µg/µl) was mixed with Fast Green and microinjected into the lumen of the NT at the flank level of the axis. Five mm tungsten electrodes were placed on either side of the embryo. A square wave electroporator (BTX) was used to deliver one pulse of current at 17-25 V for 6 ms.

### NT collection and dissociation

Embryos were electroporated at E2.5 with either control or RARα403 vectors, together with a Msx1-Citrine plasmid. At E4, ten NTs per group (with associated mesoderm) were mechanically microdissected in PBS supplemented with Ca_2_^+^/Mg_2_^+^ and 5% fetal calf serum. NTs were incubated for 15 min at 37°C in 1.5 ml Accutase (Sigma-Aldrich, A6964) containing 30 µl Papain (Sigma-Aldrich, P3125) and 300 ng DNaseI (Sigma-Aldrich, DN25) and dissociated mechanically using 1000 and 200 µl pipettes sequentially. Cells were filtered through a 40-μm filter and resuspended in PBS containing 2% fetal calf serum. Samples were sorted using a BD FACSAria III sorter (BD Biosciences). Single cells were isolated by sequentially gating cells according to their SSC-A versus FSC-A and FSC-H versus FSC-W profiles, following standard flow cytometry practices. Dead or damaged cells were excluded following propidium iodide uptake. Citrine fluorescence was detected using a 488 nm laser; age-matched NT cells from untransfected embryos served as negative control to determine the fluorescence gate.

### scRNA-seq and analysis

Sorted fluorescent cells were loaded into the 10x Genomics Chromium Next controller. Libraries were prepared following the manufacturer's instructions (GEM Single Cell 3′ GEM, Library & Gel Bead Kit v3.1, 10x Genomics). Approximately 10,000 cells were loaded per sample. Sequencing was performed using the Illumina Nextseq500 platform (Illumina Inc.) with the following sequencing conditions: 28 bp (Read1) and 54 bp (Read2).

The Cell Ranger pipeline (Zheng et al., 2017) (v6.0.1, 10x Genomics) with default parameters was used for alignment, filtering, barcode counting and unique molecular identifier (UMI) counting. The *Coturnix coturnix japonica* 2.1 genome sequence and annotations were downloaded from NCBI (RefSeq assembly GCF_001577835.2). The gtf was filtered using Cell Ranger's mkgtf command to keep protein-coding genes and lncRNAs. The Seurat R package (Satija et al., 2015) (v4.04) was used for downstream analysis and visualization. Gene–cell matrices were filtered to remove: (1) cells with mitochondrial reads comprising >8.5% of all reads; (2) cells with <250 genes or >7500 genes; (3) cells that had <500 UMIs or >40,000 UMIs. In addition, genes detected in fewer than ten cells were excluded from the analysis (Fig. S16). Doublets were processed using the R package scDblFinder (Germain et al., 2021). After implementing these quality control measures, a total of 6164 control cells, 10,191 RARα403 cells and 16,706 genes were retained. A cluster consisting of low-quality cells (1789 cells that had a low number of detected genes and low number of UMIs) was excluded. After that filtration, 5890 control cells and 8676 RARα403 cells were retained for further analysis (see ‘Quantification and statistical analysis’ section).

### Immunohistochemistry

For immunostaining, embryos were fixed overnight at 4°C with 4% paraformaldehyde in PBS (pH 7.4), embedded in paraffin wax and serially sectioned at 8 μm. Immunostaining was performed either on whole mounts or paraffin sections, as previously described (Burstyn-Cohen and Kalcheim, 2002; Kahane and Kalcheim, 2020). Antibodies were diluted in PBS containing 5% fetal bovine serum (Biological Industries, 04-007-1A) and 1% or 0.1% Triton X-100 (Sigma-Aldrich, X-100), respectively. Antibodies used were: rabbit anti-GFP (1:1000, Invitrogen, Thermo-Fisher Scientific, A-6455), chicken anti-GFP (1:500, Novus Biologicals, NB100-1614), rabbit anti-Sox9 (1:150, Millipore, AB5535), rabbit anti-Snai2 (1:500, CST, CST9585), rabbit anti-BarHL1 (1:300, Sigma-Aldrich, HPA004809), mouse anti-neurofilament-associated (1:10, Developmental Studies Hybridoma Bank, 3A10). Cell nuclei were visualized with 125 ng/ml Hoechst 33258 (Sigma-Aldrich, 14530) diluted in PBS.

### *In situ* hybridization

For ISH, embryos were fixed in Fornoy (60% ethanol, 30% formaldehyde and 10% acetic acid) for 1 h at room temperature, embedded in paraffin wax and serially sectioned at 10 μm. Briefly, sections were treated with 1 µg/ml proteinase K, re-fixed in 4% paraformaldehyde , then hybridized overnight at 65°C with digoxigenin-labeled RNA probes (Roche, 11277073910). The probes were detected with AP coupled with anti-digoxigenin Fab fragments (Roche, 11093274910). AP reaction was developed with 4-nitro blue tetrazolium chloride (NBT; Roche, 11383213001) and 5-bromo-4-chloro-3′-indolyphosphate p-toluidine salt (BCIP; Sigma-Aldrich, B8503). In the Snai2/*RSPO1* ISH experiment, the AP reaction of *RSPO1* was developed with Fast Red (Sigma-Aldrich, F4648) for 2 h at room temperature. Importantly, all hybridizations, whether done on intact embryos at NC or RP stages, or in control versus experimental embryos, were always developed for the same time for a specific probe and experiment. The RNA probes used were produced either from a vector or using a PCR product using the KAPA2G Fast ReadyMix PCR kit, (Sigma-Aldrich, KK5101). cDNA templates were synthesized by RNA precipitation followed by reverse transcription PCR. RNAs were produced from 20 somite-stage-to-E4 quail embryos. Tissue samples were homogenized with TriFast reagent, and RNA was separated with chloroform and isopropanol. RNA probes synthesized from plasmids were: *cBAMBI* (Casanova et al., 2012), c*Ednrb2* (Nitzan et al., 2013) and *cLFNG* (Ofek et al., 2021). Primers used for synthesizing probes by PCR are listed in Table S4.

### Microscopy data acquisition

Images were taken using a DP73 (Olympus) cooled CCD digital camera mounted on a BX51 microscope (Olympus) with Uplan FL-N 20×/0.5 and 40×/0.75 dry objectives (Olympus). Confocal sections encompassing their entire thickness were photographed using a Nikon Eclipse 90i microscope with a Plan Apo 40×/1.3 or 100×/1.4 objectives (Nikon) and a D-Eclipse c1 confocal system (Nikon) at 1.0 μm increments through the *z*-axis. Images were *z*-stacked with Fiji software.

For quantification, images of control and treated sections were taken under the same conditions. For figure preparation, images were exported into Photoshop CS6 (Adobe). If necessary, the levels of brightness and contrast were adjusted to the entire image. Graphics were generated using GraphPad Prism 9.0, and figures were prepared using Photoshop and InDesign CS6.

### Quantification and statistical analysis

#### scRNA-seq analysis

The expression data was normalized and log-transformed using Seurat's ‘NormalizeData’ function. The top 2000 highly variable genes were identified using Seurat's ‘FindVariableFeatures’ function with the ‘vst’ method. Each cell was assigned a cell-cycle score using Seurat's ‘CellCycleScoring’ function and the NCBI orthologs of the G2/M and S phase marker lists from the Seurat package. Potential sources of unspecific variation in the data were removed by regressing out the mitochondrial gene proportion, UMI count and the cell cycle effect using linear models and finally by scaling and centering the residuals as implemented in the function ‘ScaleData’ of the Seurat package.

For PCA, we selected 24 principal components (PCs) for downstream analyses. Cell clusters were generated using Seurat's unsupervised graph-based clustering functions ‘FindNeighbors’ and ‘FindClusters’ (resolution=0.5). UMAPs were generated using the RunUMAP on the projected PC space. Seurat's functions ‘FeaturePlot’ and ‘DimPlot’ were used for visualization. Seurat's ‘DotPlot’ function was used to generate dot plots to visualize gene expression for each cluster. Plots were further formatted using custom R scripts with the packages ggplot2 and ggnewscale. R package version 0.4.8, and patchwork (Pedersen T, 2022, patchwork: The Composer of Plots. R package version 1.1.2; https://CRAN.R-project.org/package=patchwork). Heatmaps were produced with The R package ComplexHeatmap (Version 2.14) (Gu, 2022).

Marker genes for each cluster were identified by performing differential expression between a distinct cell cluster and the cells of the other clusters with the non-parametric Wilcoxon rank sum test (Seurat's ‘FindAllMarkers’ function). Only the control sample was used for the marker identification. Cell types were assigned manually based on the expression of classic marker genes.

In order to obtain higher clustering resolution, the RP cells (cluster 18) and the DRG-melanocyte cells (clusters 11, 19 and 20) were each re-analyzed with the workflow described above using the 500 most highly variable genes with the following parameters: 20 PCs and resolution of 0.8 for the RP cells; 19 PCs and resolution of 0.4 for the DRG melanocyte cells.

In the heatmap of selected genes presented in Fig. 7, cells were obtained using Slingshot analysis (version 2.7.0) (Street et al., 2018), and ordered by the pseudotime.

#### Testing for co-expression of marker pairs in RP and DRG-melanocytes

A marker was considered to be expressed in a cell if it had a count of at least two. The number of cells that were positive for both markers was determined for each sample. A χ^2^ test was used to test the null hypothesis that the proportions are equal (prop.test in R). *P*-values were corrected for multiple comparisons using the FDR method of Benjamini and Hochberg (Benjamini and Hochberg, 1995).

Differential expression analysis inside clusters between conditions was performed using the non-parametric Wilcoxon rank sum test (Seurat's ‘FindMarkers’ function).

Out of the 16,706 genes in our data, 4422 had uncertain function (LOC symbols; LOC plus the GeneID). Of these, 40 LOC genes that exhibited notable expression patterns were assigned names based on high similarity to orthologous genes (Table S5).

#### RNA velocity and latent time analysis

Velocyto (version 0.17.17) (La Manno et al., 2018) was used to generate count matrices, based on the spliced and unspliced reads of the RARα403-treated cells, using the ‘run10x’ option. Unspliced and spliced counts were matched to the barcodes and genes retained after filtering. scVelo (version 0.2.5), an unbiased approach that leverages the distinction of unspliced and spliced RNA transcripts from the aligned sequences, enabling determination of whether the expression of a gene is being initiated or downregulated (Bergen et al., 2020; La Manno et al., 2018), was used to compute velocities using the following options: ‘filter_and_normalize’ was run using ‘min_shared_counts’ set to 20 and ‘min_cells’ set to 80; ‘monemts’ was run with ‘n_neighbors’ set to 10. Velocities were calculated using the ‘dynamical’ mode and visualized on the PCA. Phase portrait plots and PCA colored according to the velocity were created using scVelo pl.velocity function. Latent time was calculated using scVelo ‘tl.latent_time’ function. The RNA velocity analysis was performed without regressing out the cell cycle effect.

#### Microscopy data acquisition and statistical analysis

Fluorescence intensity was quantified in 11-34 sections per embryo. The number of embryos monitored per treatment is depicted in the respective figure legends. Intensities of immunofluorescence and ISH signals were measured using Fiji (Schindelin et al., 2009). In most cases, the RP was selected as the region of interest; mean intensity was measured, and background intensity was then subtracted. Average intensity of all sections was calculated per embryo, and values were normalized with a control mean set as 1.

To monitor intensity of pTP1, a segmented line with an arbitrary thickness of 20 was drawn pursuing the curvature of the NT, with the dorsal midline in its center, and further measured along the line using Fiji. Subsequent analysis and graphics were performed using R (https://www.R-project.org/) in Rstudio (http://www.rstudio.com/). The code used and the source data are available on Mendeley Data (doi:10.17632/955g6vgx6c.1).

The distance of BarHL1^+^ cells from the dorsal midline of the NT was measured for each cell manually using Fiji, and graphs were generated using R in Rstudio. The code used is available on Mendeley Data (doi:10.17632/955g6vgx6c.1).

Results were processed with GraphPad Prism 9 and presented as scatter plots with mean±s.e.m. Data were subjected to statistical analysis using either of the following tests, as described in the respective figure legends: Student's *t*-test, Welch's *t*-test, Mann–Whitney test, one-way ANOVA with post-hoc Tukey test, Kruskal–Wallis with post-hoc Dunn's test and two-way ANOVA. All tests applied were two-tailed and a *P*-value ≤0.05 was considered significant.

## Supplementary Material



10.1242/develop.202973_sup1 Supplementary information

Table S1. Top 20 genes in all clusters. The most prominently expressed genes in the UMAP presented in Fig. 1 (20 clusters) are presented.

Table S2. Top 20 genes in the re-clustered roof plate. The roof plate cluster was subdivided into 4 sub-clusters as shown in Figure S5. And the most prominently expressed genes (20/subcluster) are presented.

Table S3. Top 20 genes in re-clustered DRG-melanocyte clusters. The peripheral clusters were subdivided into 5 sub-clusters as shown in Fig. 6. The most prominently expressed genes (20/sub-cluster) are presented.

Table S6. Source data for Fig. 2.

Table S7. Source data for Fig. 3.

Table S8. Source data for Fig. 5.

Table S9. Source data for Figs 8 and 9.

Table S10. Source data for Fig. S4.
