# Supplementary material for: Retinoic acid, an essential component of the roof plate organizer, promotes the spatiotemporal segregation of dorsal neural fates
Source: Development. 2024 Sep 30;151(19):dev202973. doi: 10.1242/dev.202973 (PMC11463963; doi:10.1242/dev.202973)
Supplement: Supplementary information [file develop-151-202973-s1.pdf]

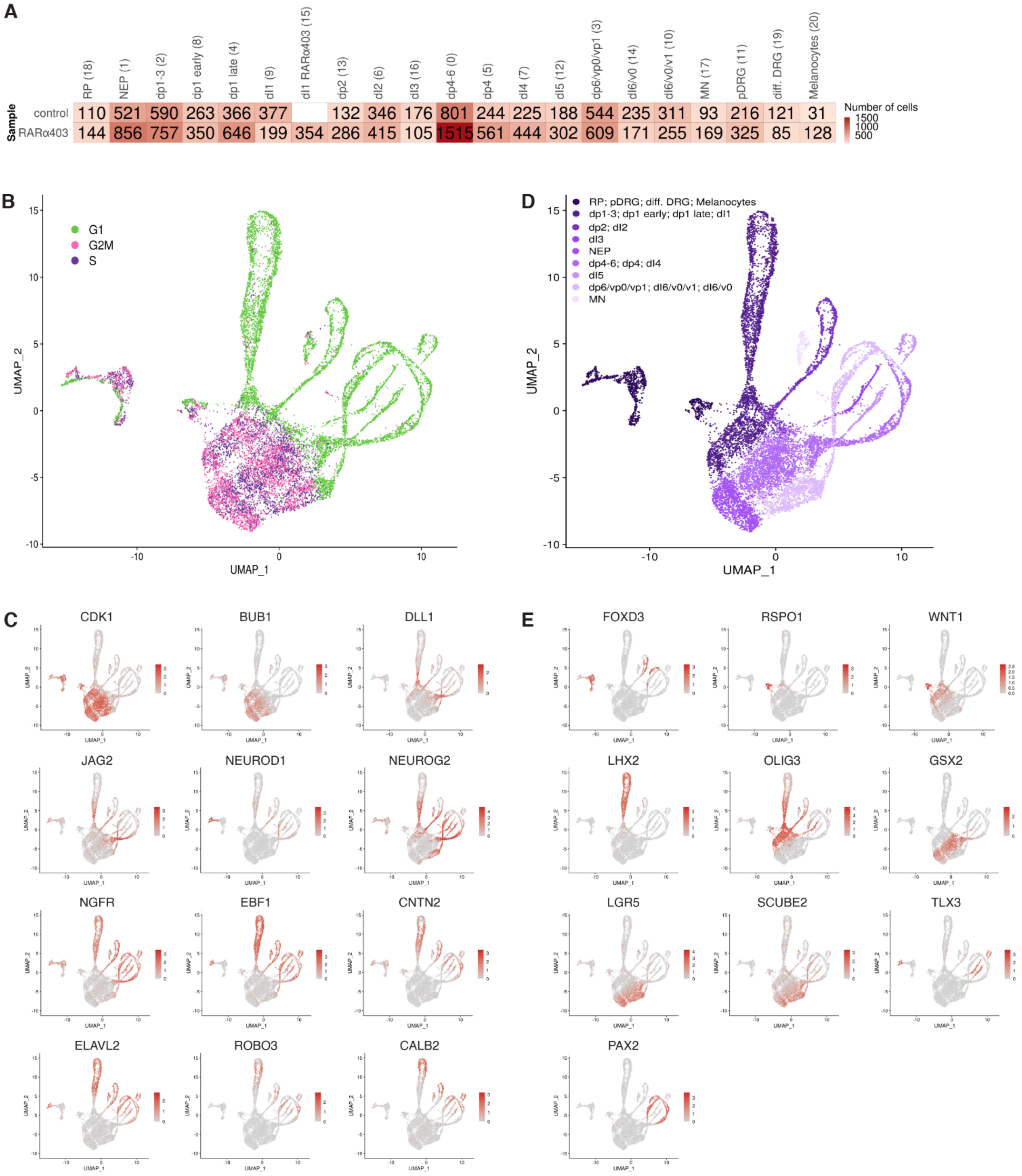
A
RP (18)
NEP (1)
dp1-3 (2)
dp1 early (8)
dp1 late (4)
dI1 (9)
dI1 RARα403 (15)
dp2 (13)
dI2 (6)
dI3 (16)
dp4-6 (0)
dp4 (5)
dI4 (7)
dI5 (12)
dp6/vp0/vp1 (3)
dI6/v0 (14)
dI6/v0/v1 (10)
MN (17)
pDRG (11)
diff. DRG (19)
Melanocytes (20)
Sample
control 110 521 590 263 366 377 132 346 176 801 244 225 188 544 235 311 93 216 121 31
RARα403 144 856 757 350 646 199 354 286 415 105 1515 561 444 302 609 171 255 169 325 85 128
Number of cells
1500
1000
500
B
G1
G2M
S
UMAP_1
UMAP_2
D
RP; pDRG; diff. DRG; Melanocytes
dp1-3; dp1 early; dp1 late; dI1
dp2; dI2
dI3
NEP
dp4-6; dp4; dI4
dI5
dp6/vp0/vp1; dI6/v0/v1; dI6/v0
MN
C
CDK1
BUB1
DLL1
JAG2
NEUROD1
NEUROG2
NGFR
EBF1
CNTN2
ELAVL2
ROBO3
CALB2
E
FOXD3
RSPO1
WNT1
LHX2
OLIG3
GSX2
LGR5
SCUBE2
TLX3
PAX2

Fig. S1. Temporal and spatial organization of cells in the UMAP. Related to Figure 1. A) Comparison between the number of cells per cluster, in both control and RARα403 samples. B) A UMAP colored by cell cycle phase. Note that dividing progenitors are mainly located in the octopus head. C) UMAP plots colored by the expression of selected genes corresponding to successive stages in neuronal development. D) NT cell types colored from dorsal to ventral (dark to light purple, respectively). NC derivatives are grouped with the RP. E) Selected genes that characterize various cell types in the dorsal to ventral extent of the NT.

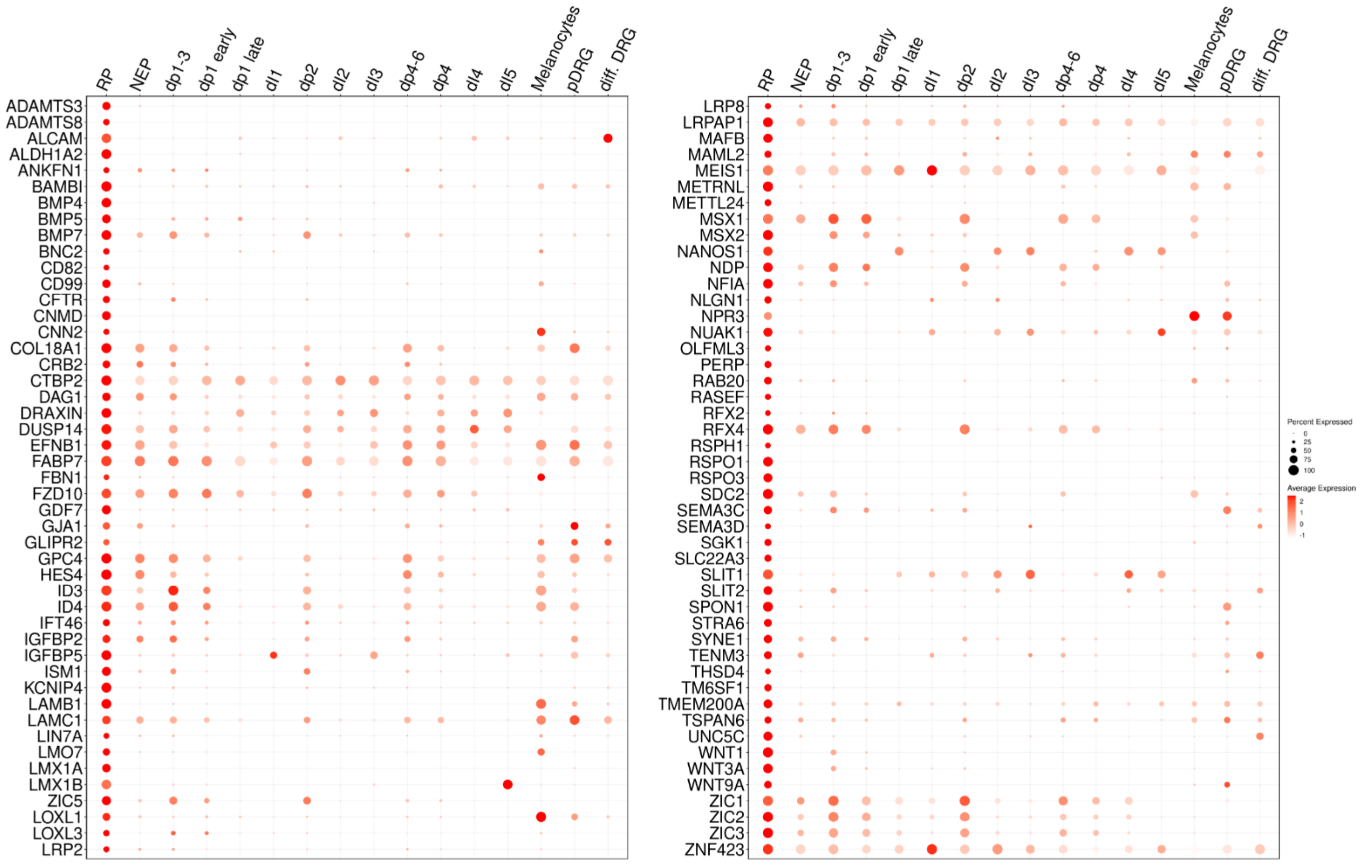

Fig. S2. scRNA-seq reveals an array of novel RP markers. Related to Fig. 1. Dot plot of the control sample depicting expression of RP markers compared to other clusters (expression fold change >1.4 relative to other clusters).

Development • Supplementary information

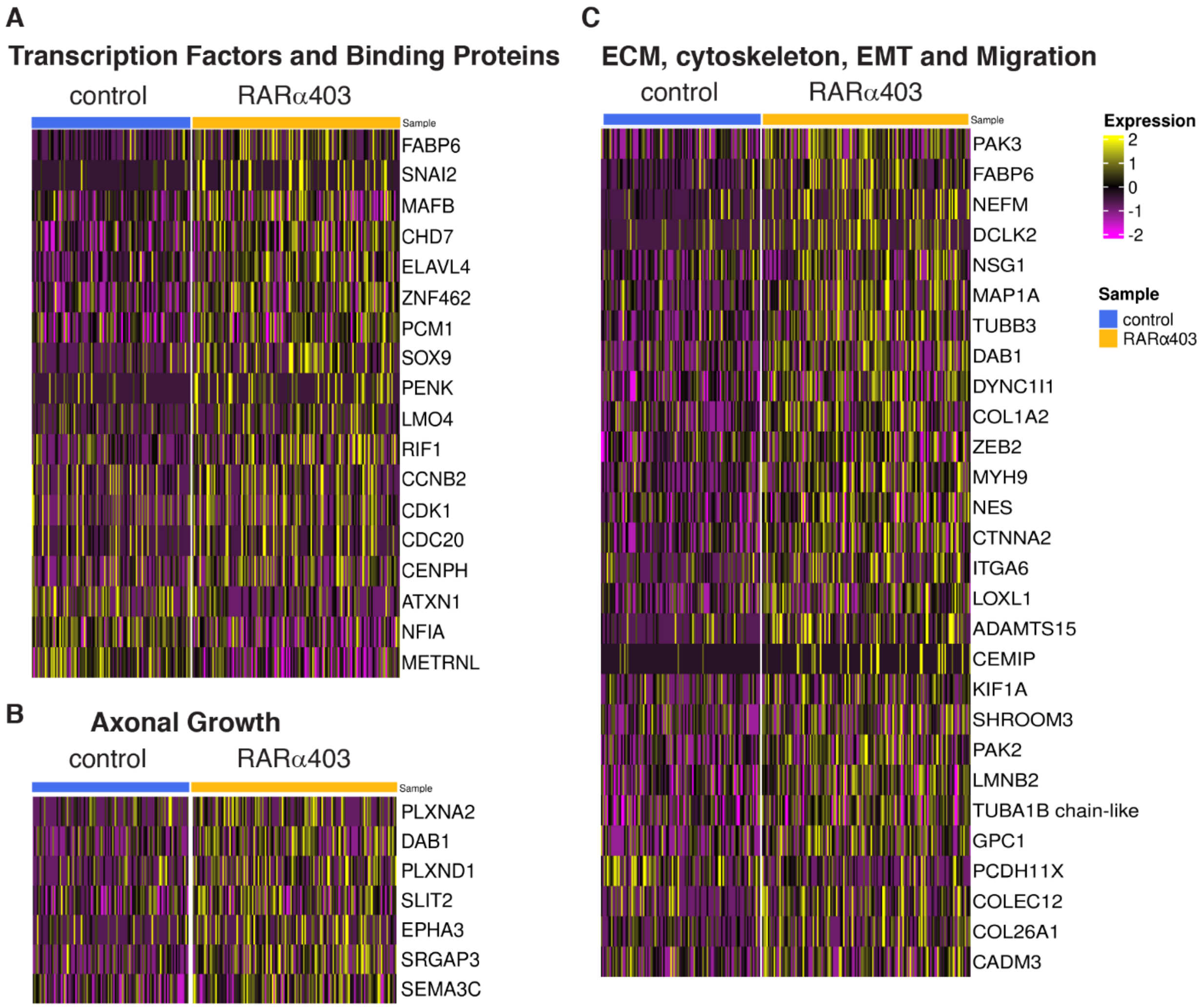

Fig. S3. Differential gene expression in RP reveals changes in transcription factors, adhesive properties and axonal growth. Related to Fig. 3. Differential gene expression (selected from genes with minimum linear fold change ±1.3, p-value<0.05) between RARα403-treated and control RP clusters, corresponding to the following categories: A) transcription factors and binding proteins, B) Axonal growth, and C) ECM, cytoskeleton, EMT and migration.

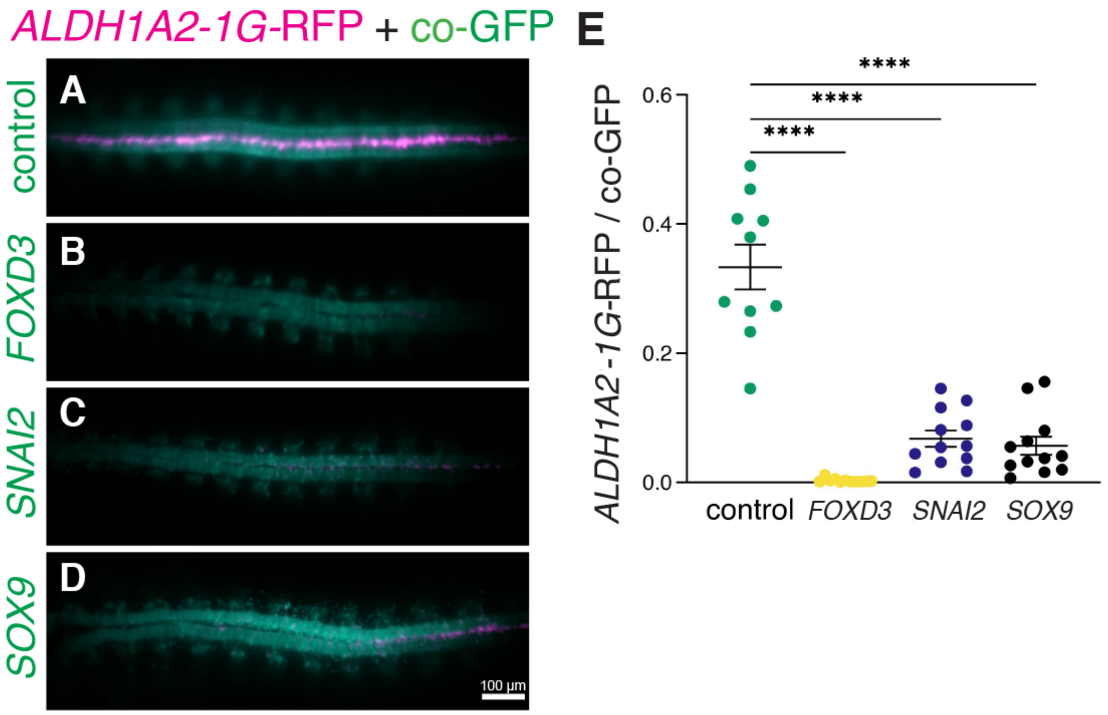

Fig. S4. NC and RP genes stand in a mutually repressive interaction. Related to Fig. 4. Embryos were electroporated at E2.5 with either a control plasmid or a plasmid expressing *FOXD3*, *SNAI2* or *SOX9*, along with GFP to mark electroporation efficiency and location. RFP is driven by the RP-specific *ALDH1A2* enhancer. Note significant reduction of ALDH1A2 activity in treated RPs. Whole mount embryos were photographed at E4 (A-D) and intensity of RFP/GFP was quantified (E). ****$p < 0.0001$, Welch's t-test. See Table S10 for source data.

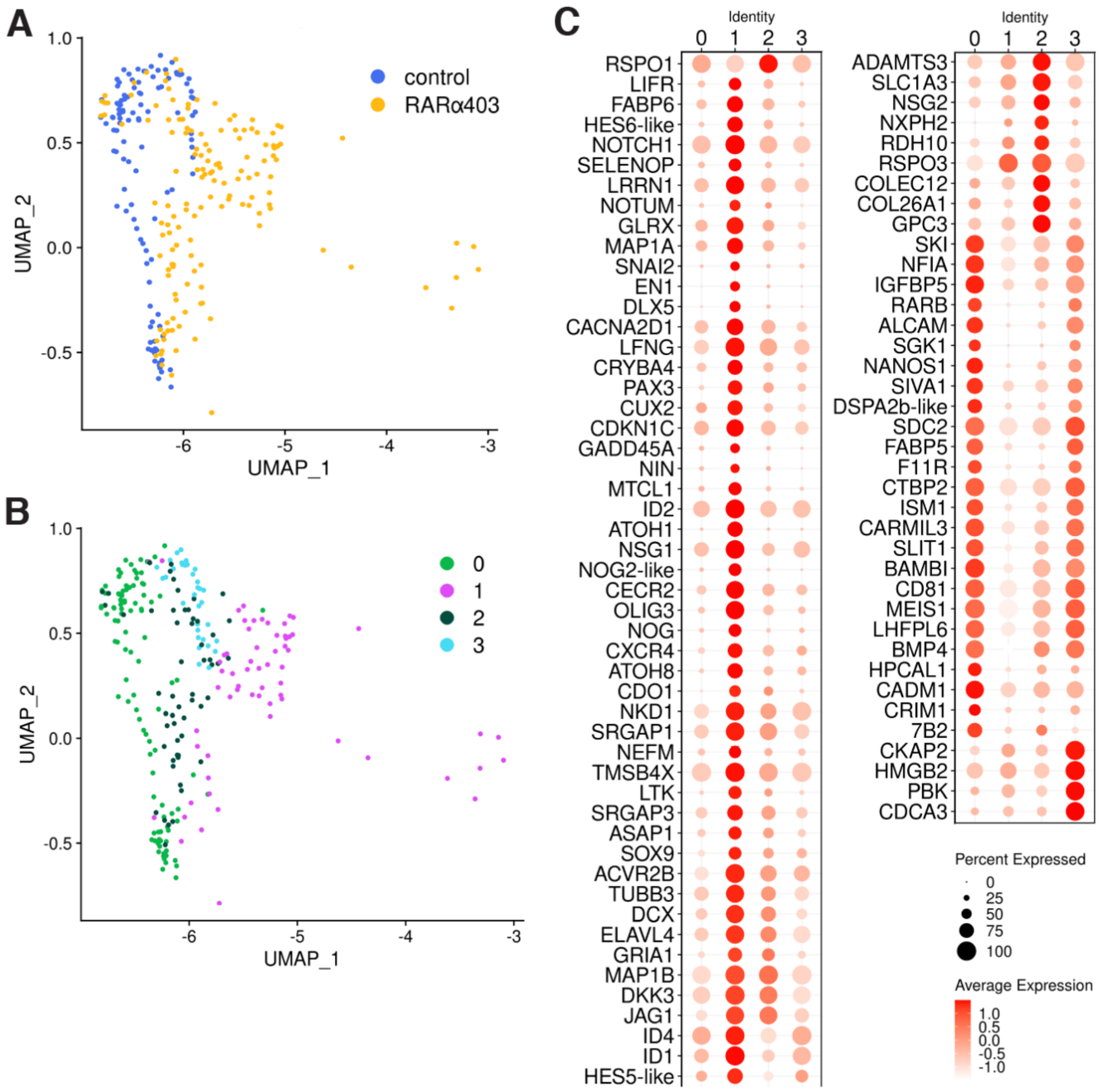

Fig. S5. Re-clustering of the RP reveals a subcluster with a mixed identity. Related to Fig. 4.
A) UMAP of the RP cluster, colored by sample. B) Re-clustering of the RP yields four subclusters. C) Dot plot depicting marker genes differentially expressed within the RP subclusters.

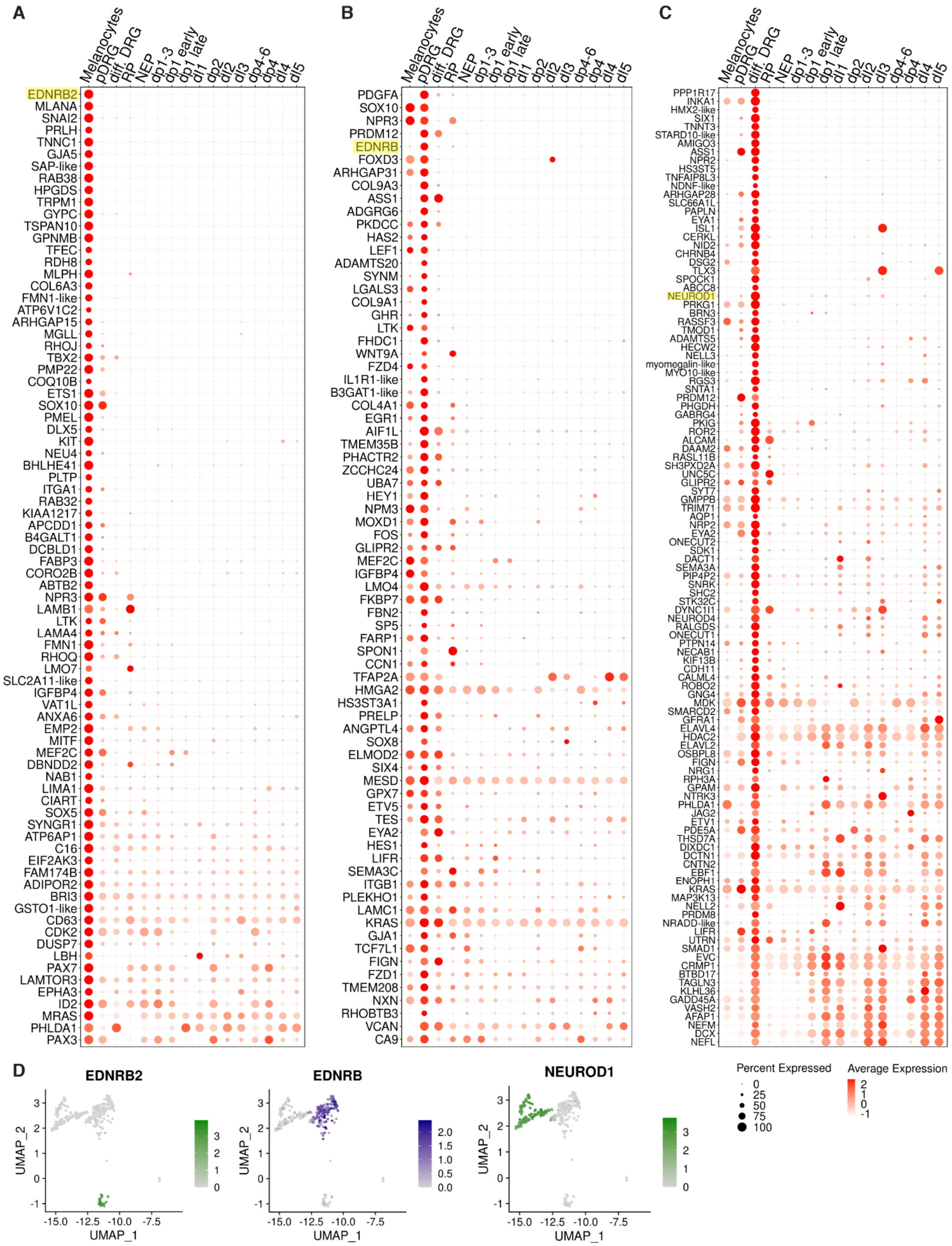

Development • Supplementary information

Fig. S6. Gene expression in NC-derived peripheral clusters. Related to Figure 6. A-C) Dot plots of the control sample depicting marker gene expression in peripheral clusters (A, melanocytes; B, pDRG; C, diff. DRG) compared to other clusters. Expression fold change >1.4 relative to other clusters. Marked in yellow are genes visualized in D. D) UMAPs demonstrating the expression of EDNRB2 and EDNRB (guidance receptors for lateral vs. ventral migration, respectively), and NEUROD1, a neuronal gene. Note the clear distinction between domains of expression.

Development • Supplementary information

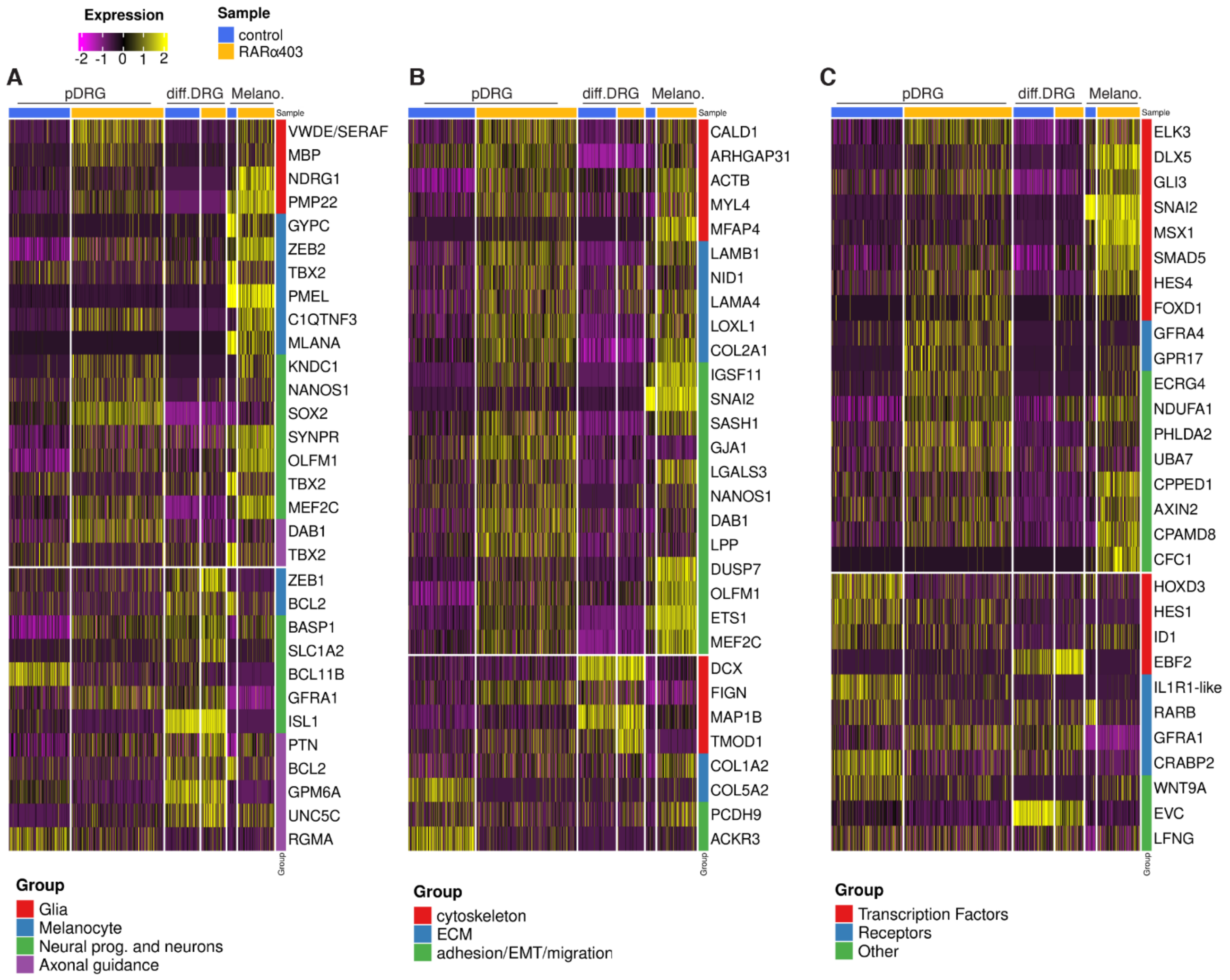

Fig. S7. Differential gene expression in NC-derived peripheral clusters. Related to Fig. 6. Differential gene expression between RARα403-treated and control peripheral clusters, corresponding to A) Glia, melanocyte, neural and axonal guidance, B) Cytoskeleton, ECM, adhesion/EMT/migration and C) Transcription factors, receptors and other genes categories. Selected genes had a minimum linear fold change ±1.3, p-value and adjusted p- value <0.05.

Development • Supplementary information

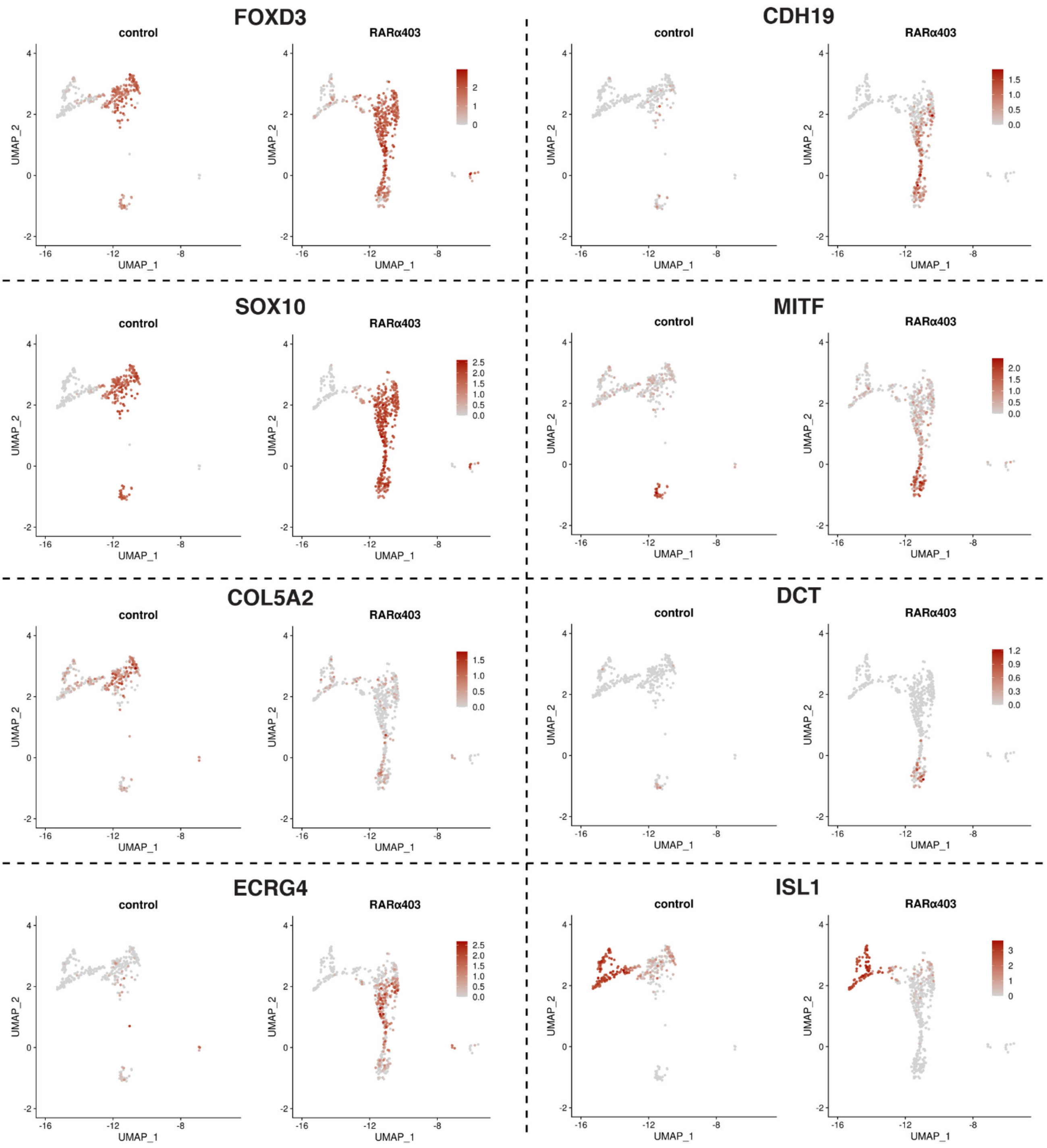

Development • Supplementary information

Fig. S8. Expression of selected genes in control and RARα403-treated peripheral clusters. Related to Fig. 6. Feature plots showing expression of pDRG (cluster 0 in Fig. 5B), bridge (cluster 1) melanocyte (cluster 3) and neuronal markers (clusters 2,4) in the peripheral clusters. The progenitor genes FOXD3 and SOX10 are upregulated in the bridge of treated embryos. COL5A, a pDRG marker, is downregulated under treated conditions whereas ECRG4 (augurin) and CDH19 (formerly cadherin 7) are either absent or expressed in few control cells, respectively, and upregulated in the bridge. MITF and DCT mark melanocytes, and ISL1 marks sensory neurons.

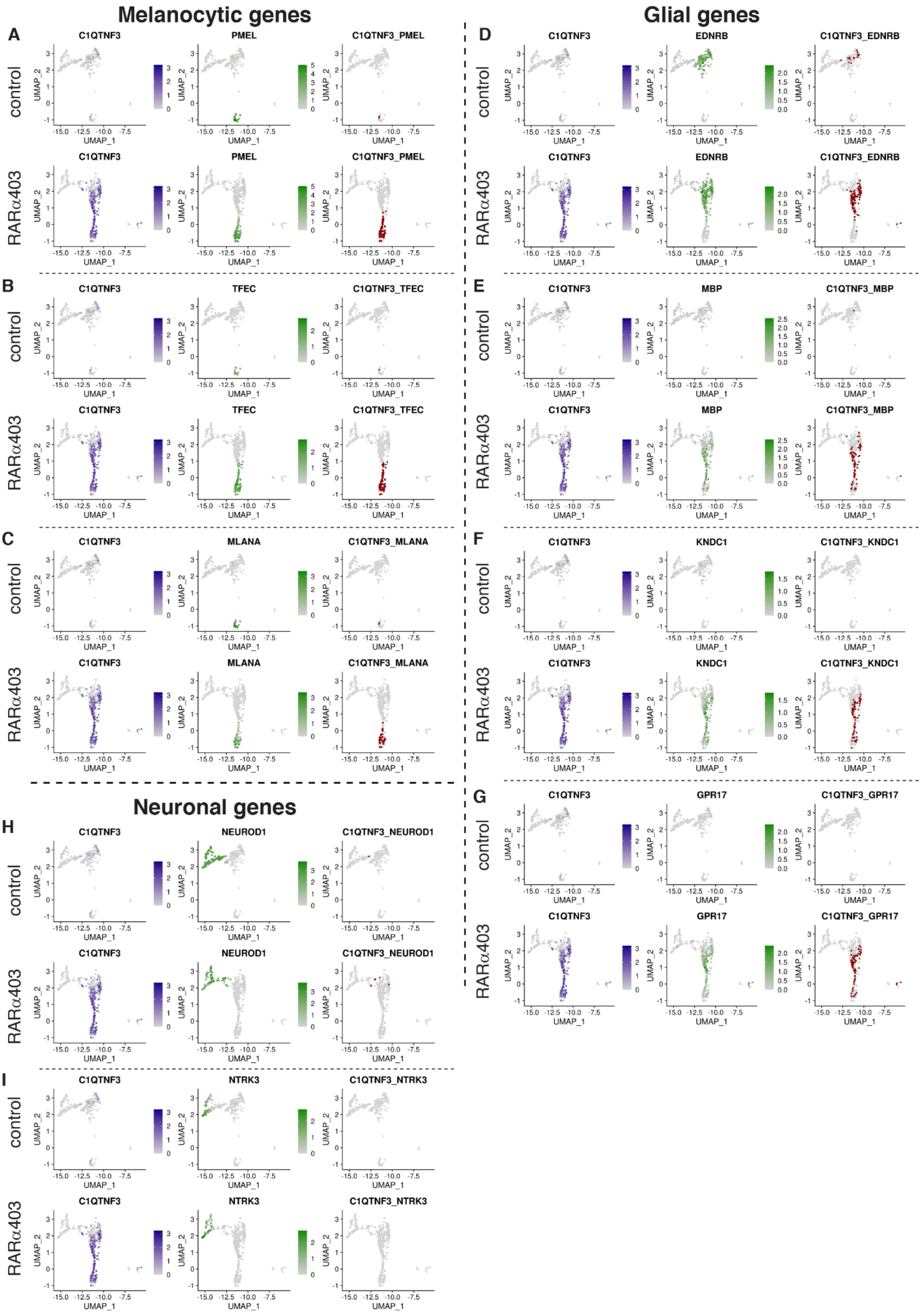
Melanocytic genes
Glial genes
Neuronal genes
A
B
C
D
E
F
G
H
I
control
RARα403
C1QTNF3
PMEL
C1QTNF3_PMEL
TFEC
C1QTNF3_TFEC
MLANA
C1QTNF3_MLANA
EDNRB
C1QTNF3_EDNRB
MBP
C1QTNF3_MBP
KNDC1
C1QTNF3_KNDC1
GPR17
C1QTNF3_GPR17
NEUROD1
C1QTNF3_NEUROD1
NTRK3
C1QTNF3_NTRK3
UMAP_1
UMAP_2

Fig. S9. ***C1QTNF3*-expressing bridge cells co-express glia and melanocyte, but not neuronal genes. Related to Fig. 8.** Co-expression of *C1QTNF3* with melanocytic (A-C), glial (D-G) and neuronal (H-I) genes in both RARα403 and control samples, visualized on UMAPs of the peripheral clusters. Cells which had at least two reads/cell of the genes of interest are considered double-positive and stained red in the right column. A-G) Note the negligible proportion of double-positive cells in the control sample, as opposed to significant co-expression of *C1QTNF3* with glia and melanocyte genes in the bridge of the treated sample. H-I) Virtually no co-expression is detected between *C1QTNF3* and neuronal genes.

Development • Supplementary information

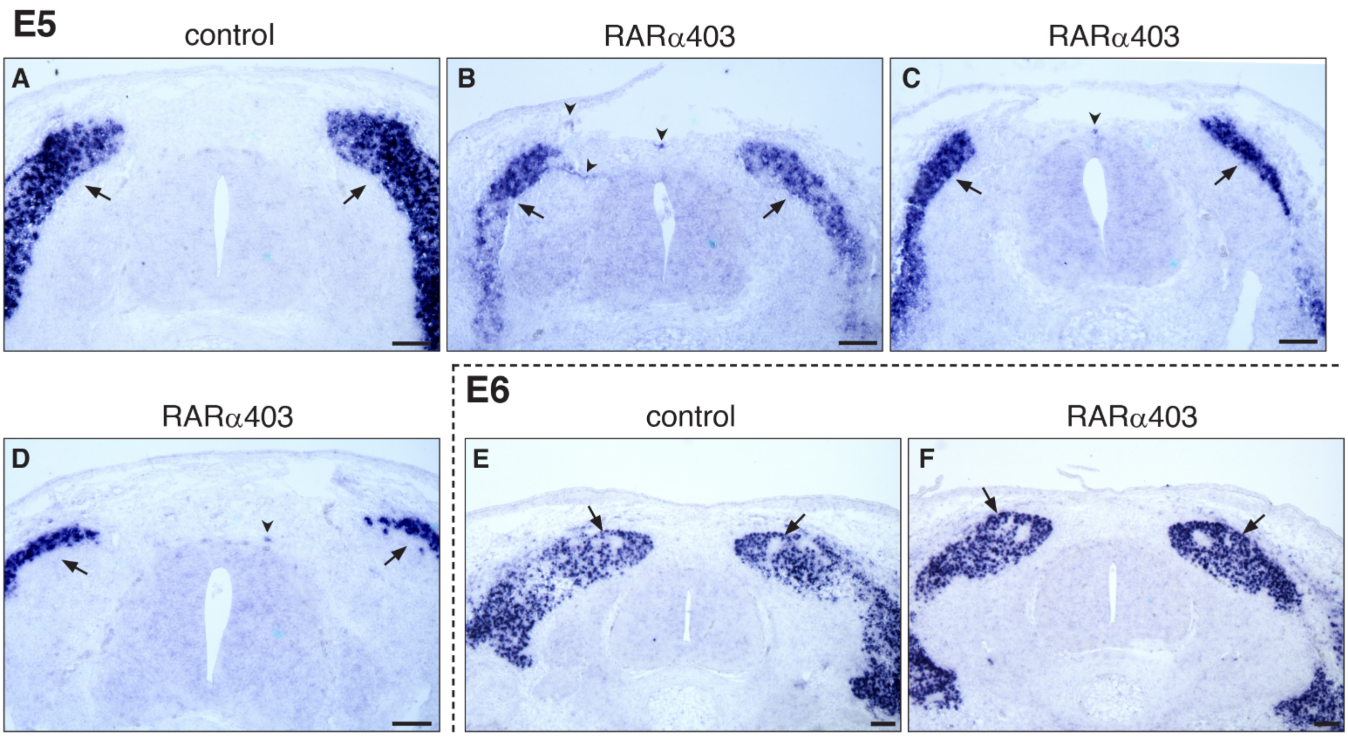

Fig. S10. Expression of *C1QTNF3* in control and RARα403-treated embryos at E5 and E6. Related to Fig. 9. Embryos were electroporated with control PCAGG or RARα403 at E2.5, fixed at E5 (A-D) or E6 (E,F) and in-situ hybridized for expression of *C1QTNF3*. Arrows indicate normal expression in myotomes of both control and treated embryos. Arrowheads in B-D highlight positive cells in the mesoderm adjacent to the dorsal NT, present exclusively in the treated embryos. At E6 (E,F) no crest-derived *C1QTNF3*$^{+}$ cells were detected any longer. N=3 embryos for control groups, and N=4 for RARα403-treated groups. Scale bar, 100 μm.

Development • Supplementary information

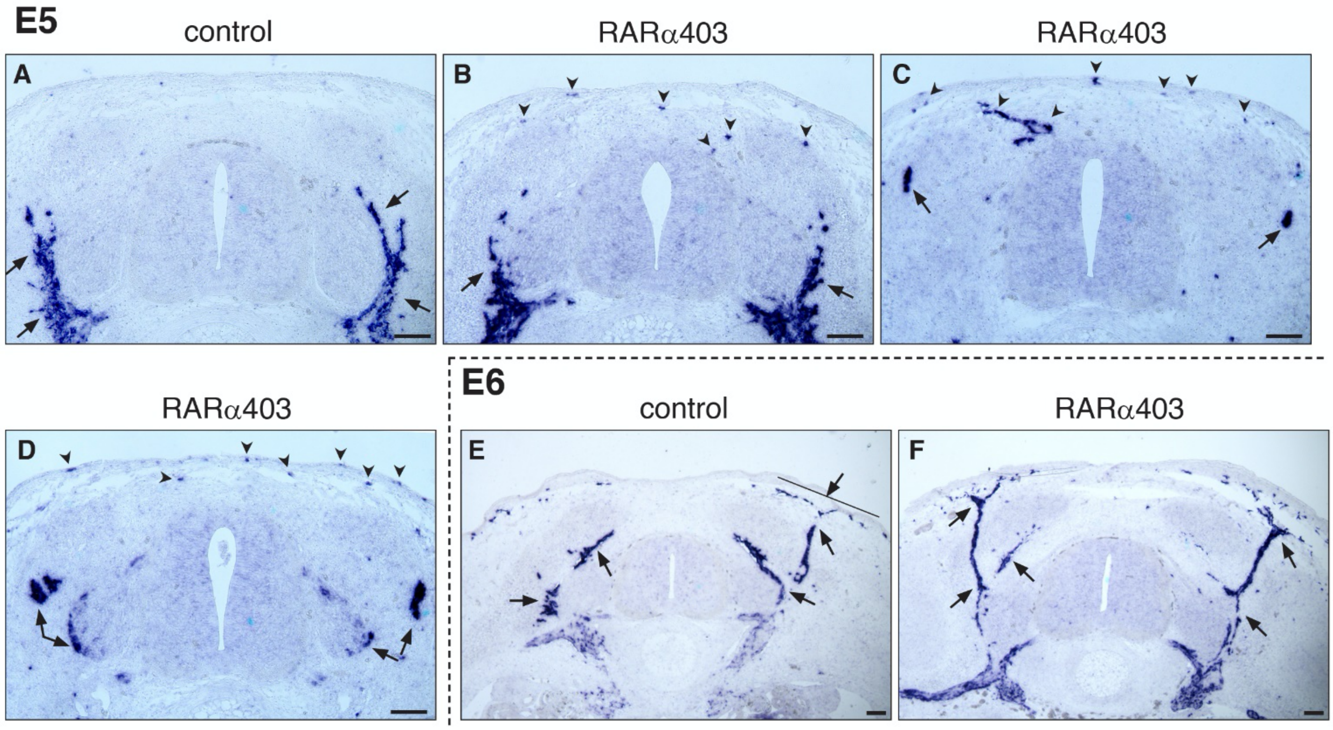

Fig. S11. Expression of *SERAF* in control and RARα403-treated embryos at E5 and E6. Related to Fig. 9. Embryos were electroporated with control PCAGG or RARα403 at E2.5, fixed at E5 (A-D) or E6 (E,F) and in-situ hybridized for expression of *SERAF*. Arrows indicate normal expression along peripheral nerves of both control and treated embryos. Arrowheads in B-D highlight numerous positive cells scattered throughout the mesoderm dorsal to the neural tube, in dermis and in the epidermis corresponding to sites populated by melanocytes and present exclusively in the treated embryos. At E6 (E,F) no differences between treatments were detected, with *SERAF*$^{+}$ cells present along ventral and dorsal rami. Note as well the presence of positive cells in both treatments that accompany defasciculating nerve terminals in the dorsal dermis (bar in D and a similar pattern in E). N=3 embryos for control groups, and N=4 for RARα403-treated groups. Scale bar, 100 μm.

Development • Supplementary information

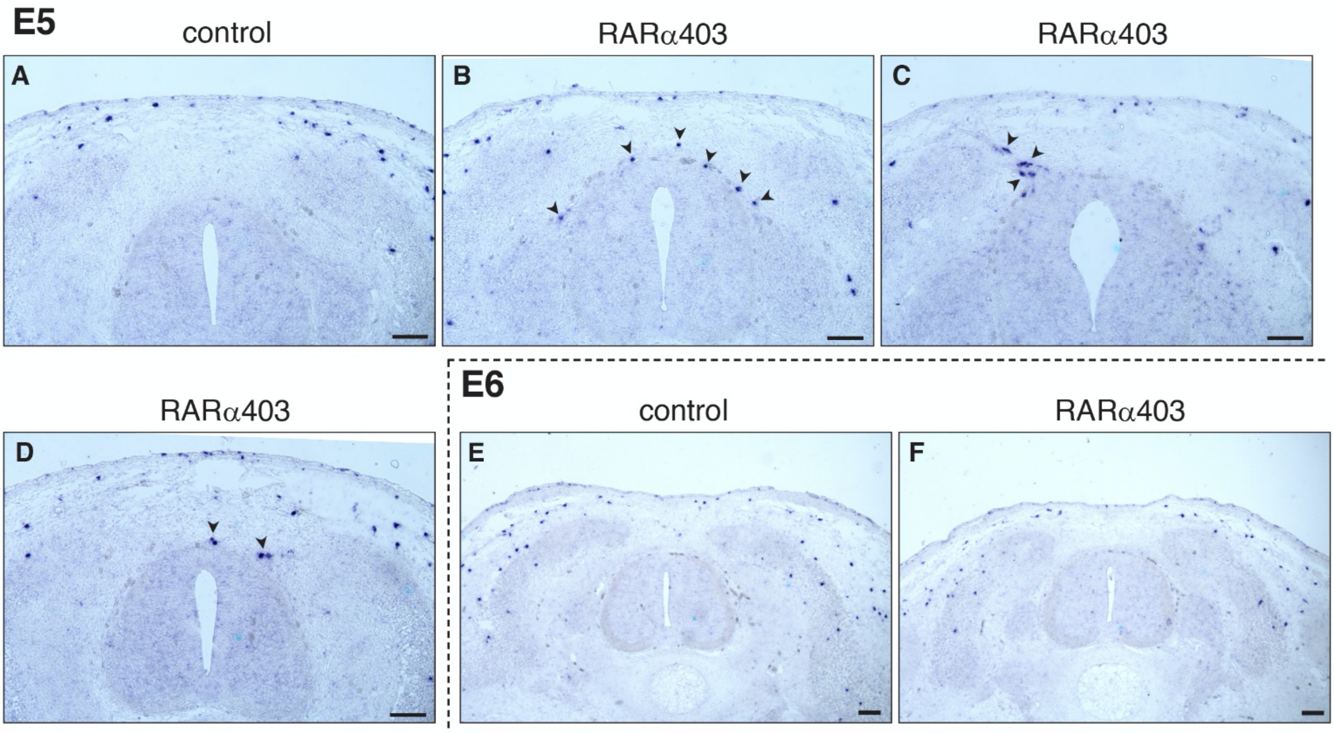

Fig. S12. Expression of *EDNRB2* in control and RARα403-treated embryos at E5 and E6. Related to Fig. 9. Embryos were electroporated with control PCAGG or RARα403 at E2.5, fixed at E5 (A-D) or E6 (E,F) and in-situ hybridized for expression of *EDNRB2*. In controls at E5, note *EDNRB2*$^{+}$ cells in dermis and epidermis corresponding to the localization of normal melanocytes (A). In treated embryos (B-D) additional cells are present adjacent to the dorsal NT (B,D, arrowheads) and also accompany nerve fibers likely extending from dorsal interneurons outside the confines of the NT (arrowheads in C, and see also Fig. 3L,M). At E6, *EDNRB2*$^{+}$ cells are scattered throughout the dermis and epidermis in both control and treated cases (E,F). N=3 embryos for control groups, and N=4 for RARα403-treated groups. Scale bar, 100 µm.

Development • Supplementary information

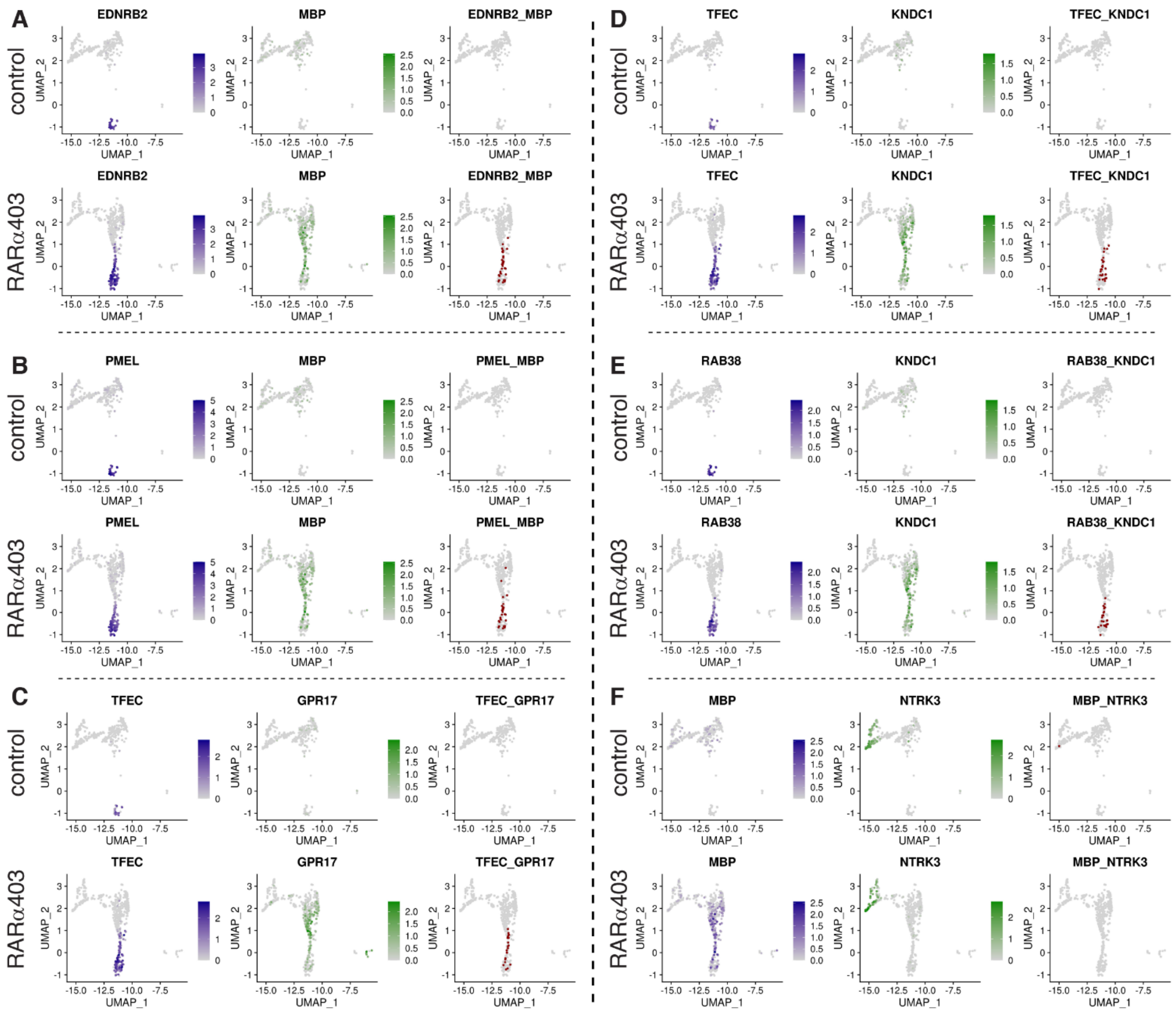

Fig. S13. Bridge cells co-express glia and melanocyte genes. Related to Fig. 11. Co- expression of glia/melanocyte (A-E) and neuronal (F) genes in both control and RARα403 samples, visualized on UMAPs of the peripheral clusters. Cells which had at least two reads/cell of the genes of interest are considered double-positive and stained red in the right column. A-E) Note the negligible proportion of double-positive cells in the control sample, as opposed to significant co-expression of glia and melanocyte genes in the bridge of the treated sample. F) No co-expression is detected between glia (*MBP*) and neuronal (*NTRK3*) genes.

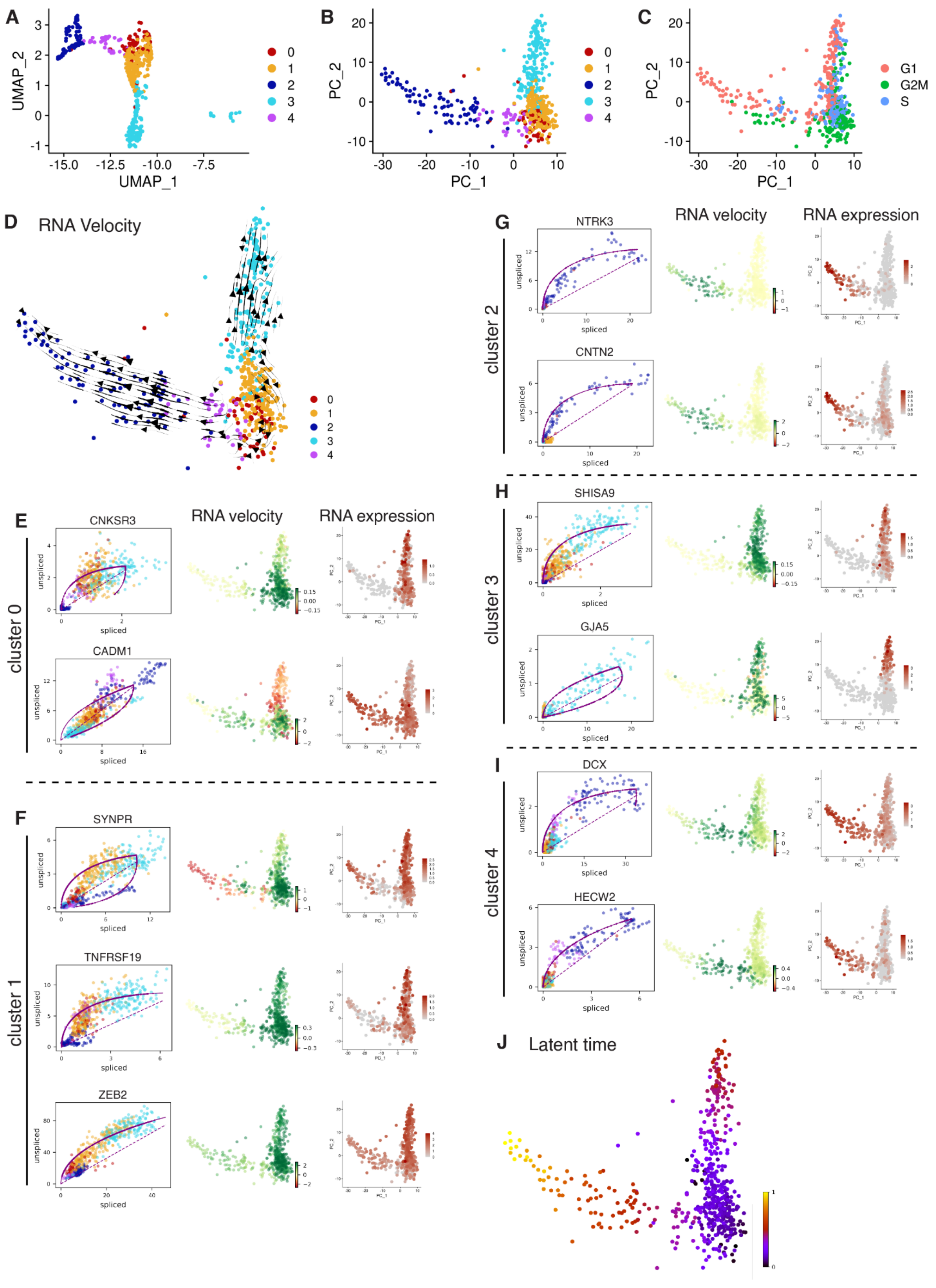
A
B
C
UMAP_2
UMAP_1
PC_2
PC_1
0
1
2
3
4
G1
G2M
S
D
RNA Velocity
E
cluster 0
CNKSR3
CADM1
RNA velocity
RNA expression
unspliced
spliced
F
cluster 1
SYNPR
TNFRSF19
ZEB2
G
cluster 2
NTRK3
CNTN2
H
cluster 3
SHISA9
GJA5
I
cluster 4
DCX
HECW2
J
Latent time

Fig. S14. Velocity analysis reveals bridge cells as an origin for melanocytes and DRG cells. Related to Fig. 11. A-B) Re-clustering of the peripheral cell types projected on UMAP and PCA, respectively. C) PCA colored by the cell cycle phase (predicted using Seurat's CellCycleScoring function). D) RNA velocity vectors projected on a PCA of peripheral clusters. E-I) Examples of specific genes with high velocity in different subclusters. Left, phase portraits of spliced versus unspliced transcripts, with a dotted line indicating the steady state of transcription and a fitted curve indicating the learned dynamics; middle, PCA colored by the velocity; right, RNA expression level. J) Latent time analysis of peripheral clusters. Earliest time point is depicted in black/purple and latest in yellow.

Development • Supplementary information

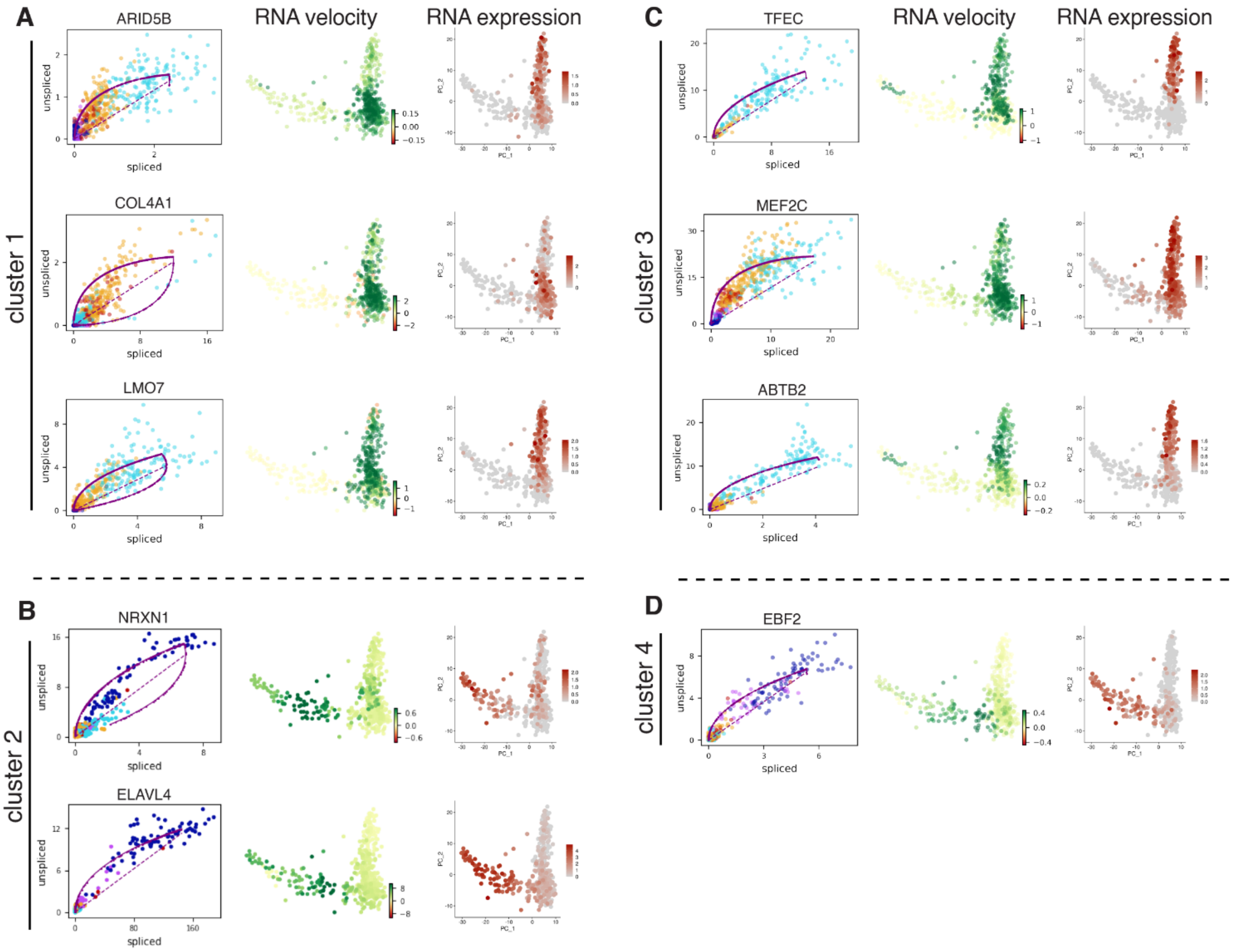

Fig. S15. Velocity analysis reveals bridge cells as an origin of melanocytes and DRG cells. Related to Fig. 11. Additional examples of specific genes with high velocity in different subclusters. Left, phase portraits of spliced versus unspliced transcripts, with a dotted line indicating the steady state of transcription and a fitted curve indicating the learned dynamics; middle, PCA colored by velocity; right, RNA expression level.

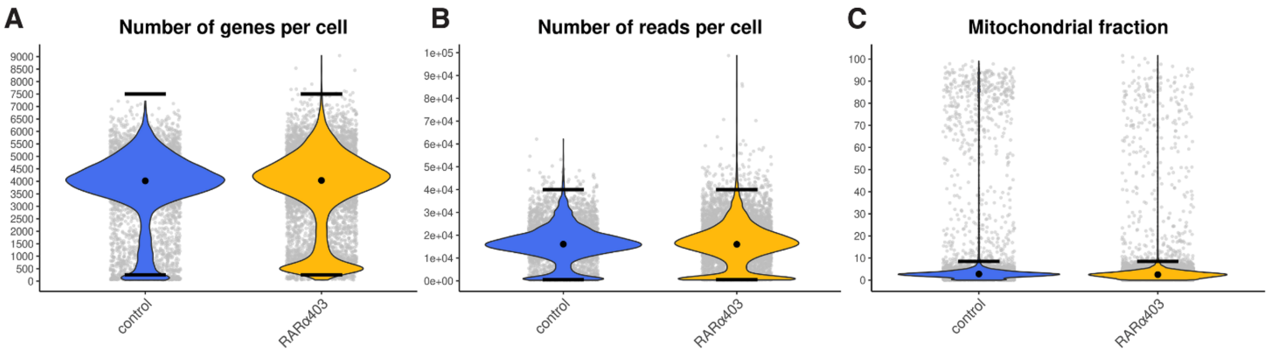

Fig. S16. Quality controls of the scRNA-seq. A) Violin plot showing the number of genes detected in each cell. Cells with less than 250 genes or more than 7500 genes (horizontal lines) were filtered out. **B)** Violin plot showing the number of UMIs detected in each cell. Cells that had less than 500 UMIs or more than 40,000 UMIs (horizontal lines) were filtered out. **C)** Violin plot showing the fraction of reads that mapped to mitochondrial genes. Cells that contained more than 8.5% (horizontal lines) of the reads aligned to mitochondrial genes were filtered out.

Development • Supplementary information

Table S1. Top 20 genes in all clusters. The most prominently expressed genes in the UMAP presented in Fig. 1 (20 clusters) are presented.

Available for download at
https://journals.biologists.com/dev/article-lookup/doi/10.1242/dev.202973#supplementary-data

Table S2. Top 20 genes in the re-clustered roof plate. The roof plate cluster was subdivided into 4 sub-clusters as shown in Figure S5. And the most prominently expressed genes (20/sub-cluster) are presented.

Available for download at
https://journals.biologists.com/dev/article-lookup/doi/10.1242/dev.202973#supplementary-data

Table S3. Top 20 genes in re-clustered DRG-melanocyte clusters. The peripheral clusters were subdivided into 5 sub-clusters as shown in Fig. 6. The most prominently expressed genes (20/sub-cluster) are presented.

Available for download at
https://journals.biologists.com/dev/article-lookup/doi/10.1242/dev.202973#supplementary-data

Table S4. Primers used for generating probes for ISH

Gene	Forward primer	Reverse primer
qDLX5	GTTTGACAGAAGGGTCCCCA	CACTTTCTTTGGCTTGCCGT
qKNDC1	AGTTTGGCTGCGAATGGGAA	GCTCTAGGAAGGCTGGTACG
qSERAF	CCCTCAAGGTTGCAGGAATA	AACCGAGACATAGCAGTTCT
qC1QTNF3	AGTTTGGCTGCGAATGGGAA	GCTCTAGGAAGGCTGGTACG
qNANOS1	GCTTCATCACATTGCTCCAG	CTTCTGTGTTTGTAGCGAGC
qRSPO1	AAACCACCGGTCTCTGTGTC	AGCAGGAGGGAAGGAAGAAG
qRSPO3	TGCATCCTAACGTGAGCCAG	CACGGACTCCACTCACTAGC
qADAMTS8	TGATGGCCCCACTCTTTGTC	AGCCGTACTTGGATCGGTTG
qALCAM	GCGTCAAAACACGTAGACAA	CTTTAAGGCAGCGGATAACG
qDLL1	TACTGCACTGAGCCGATTTG	CACCATCAGGGTTGTCAGTG

Development • Supplementary information

Table S5. A list of identified LOC genes

LOC	Gene
LOC107312830	EDNRB2
LOC107307993	ZIC5
LOC107317839	HES6-like
LOC107308537	SFRP2-like
LOC107320998	noggin2-like/NOG2-like
LOC107323479	HES5-like
LOC107309618	TUBB2 chain-like
LOC107325741	TUBA1B chain-like
LOC107310329	FABP5
LOC107314969	7B2
LOC107314285	DSPA2b-like
LOC107315152	CECR2
LOC107306834	IL1R1-like
LOC107320417	B3GAT1-like
LOC107313875	HMX2-like
LOC107320292	STARD10-like
LOC107315537	NDNF-like
LOC107310125	DSG2
LOC107312758	BRN3/POU3F3
LOC107308986	NELL3
LOC107317105	myomegalin-like
LOC107316896	MYO10-like

Development • Supplementary information

LOC107312875	GABRG4
LOC107308730	NRADD-like
LOC107324519	SAP-like
LOC107314961	FMN1-like
LOC107321228	SLC2A11-like
LOC107313434	C16
LOC107309133	VWDE/SERAF
LOC107316089	KNDC1
LOC107307182	MFAP4
LOC107308928	ZEB1
LOC107306284	CFC1
LOC107314568	PHLDA2
LOC107316274	COE3-like
LOC107316810	HEG1
LOC107319512	B-cadherin-like
LOC107324632	PTPRV
LOC107317441	SEPP1B-like
LOC107324454	S100A6

Development • Supplementary information

Table S6. Source data for Fig. 2.

Available for download at
https://journals.biologists.com/dev/article-lookup/doi/10.1242/dev.202973#supplementary-data

Table S7. Source data for Fig. 3.

Available for download at
https://journals.biologists.com/dev/article-lookup/doi/10.1242/dev.202973#supplementary-data

Table S8. Source data for Fig. 5.

Available for download at
https://journals.biologists.com/dev/article-lookup/doi/10.1242/dev.202973#supplementary-data

Table S9. Source data for Figs 8 and 9.

Available for download at
https://journals.biologists.com/dev/article-lookup/doi/10.1242/dev.202973#supplementary-data

Table S10. Source data for Fig. S4.

Available for download at
https://journals.biologists.com/dev/article-lookup/doi/10.1242/dev.202973#supplementary-data
