# Supplementary material for: Retinoic acid, an essential component of the roof plate organizer, promotes the spatiotemporal segregation of dorsal neural fates
Source: Development. 2024 Sep 30;151(19):dev202973. doi: 10.1242/dev.202973 (PMC11463963; doi:10.1242/dev.202973)
Supplement: Supplementary file 1 [file develop-151-202973_review_history.pdf]

Retinoic acid, an essential component of the roof plate organizer, promotes the spatio-temporal segregation of dorsal neural fates

Dina Rekler, Shai Ofek, Sarah Kagan, Gilgi Friedlander and Chaya Kalcheim
DOI: 10.1242/dev.202973

Editor: Debra Silver

Review timeline
Original submission: 18 April 2024
Editorial decision: 28 May 2024
First revision received: 24 July 2024
Accepted: 26 August 2024

Original submission

First decision letter

MS ID#: DEVELOP/2024/202973

MS TITLE: Retinoic acid, an essential component of the RP organizer, promotes the spatio-temporal segregation of dorsal neural fates

AUTHORS: Dina Rekler, Shai Ofek, Sarah Kagan, Gilgi Friedlander, and Chaya Kalcheim

I have now received all the referees' reports on the above manuscript, and have reached a decision. The referees' comments are appended below, or you can access them online: please go to BenchPress and click on the 'Manuscripts with Decisions' queue in the Author Area.

As you will see, the referees express considerable interest in your work, but have some significant criticisms and recommend a substantial revision of your manuscript before we can consider publication. This includes suggestions for bioinformatic analysis, inclusion of in situs, as well as fleshing out of points to address concerns about novelty (raised by reviewer 3).

If you are able to revise the manuscript along the lines suggested, I will be happy receive a revised version of the manuscript. Your revised paper will be re-reviewed by one or more of the original referees, and acceptance of your manuscript will depend on your addressing satisfactorily the reviewers' major concerns. Please also note that Development will normally permit only one round of major revision. If it would be helpful, you are welcome to contact us to discuss your revision in greater detail. Please send us a point-by-point response indicating your plans for addressing the referees' comments, and we will look over this and provide further guidance.

Please attend to all of the reviewers' comments and ensure that you clearly highlight all changes made in the revised manuscript. Please avoid using 'Tracked changes' in Word files as these are lost in PDF conversion. I should be grateful if you would also provide a point-by-point response detailing how you have dealt with the points raised by the reviewers in the 'Response to Reviewers' box. If you do not agree with any of their criticisms or suggestions please explain clearly why this is so.

Reviewer 1

Advance summary and potential significance to field

© 2024. Published by The Company of Biologists under the terms of the Creative Commons Attribution License (https://creativecommons.org/licenses/by/4.0/).

The authors investigate the role of retinoic acid (RA) in the formation and differentiation of cell types in the developing neural tube, focusing particularly on the segregation of neural crest cells, roof plate and the rest of the dorsal central nervous system cells. The authors employed in-ovo gene misexpression and single-cell RNA profiling to explore how RA influences the spatial and temporal segregation of cell fates. The findings indicate that RA is crucial for the correct separation between NC and dorsal CNS cells forming the roof plate (RP). The study reveals that RA might modulate this process by suppressing BMP and Wnt signaling pathways, which are essential for the initiation of the epithelial-mesenchymal transition in NC cells. The observation of a hybrid cell phenotype in the dorsal neural tube in case of RA inhibitor treatment is the pivotal observation, which is most interesting to me. Indeed the authors convincingly show that the absence of RA signaling disrupts this organized segregation, leading to an intriguing mixing of cell type features also including the invasion of RP by dI1 interneurons, which normally should not occur. The study also identifies that Notch signaling, essential for RP and dI1 interneuron formation, is restricted by RA to the boundary regions between these cells, facilitating proper spatial separation.

The last but not the least, the authors show that RA signaling influences the processes of fate selection in the neural crest lineage leading to the expansion of melanocyte-glial progenitors, most likely Schwann cell precursors and satellite glial cells that become biased towards melanocyte lineage in the absence of RA signaling. This is the second most interesting results to me.
Overall, the study is well done and is technically sound in a general way though it requires some technical tuning prior to publishing.

Comments for the author

Overall, the study is well done and is technically sound in a general way though it requires some technical tuning prior to publishing.

1. The single cell data require the inclusion of a technical supplementary figure showing quality controls such as numbers of genes per cell, numbers of reads per cell, doublet detection and removal, mitochondrial fraction, top 20 genes per every cluster (unbiasedly define) for every umap embedding.

2. Figure 1E,F: When it comes to showing compositional differences between control and experimental condition (RA inhibition), the current standards in the filed suggest doing several sequencing rounds (3-4 per condition, could be smaller batches) to calculate the significance of the effect, as single cell datasets are prone to dropouts and other technical problems. In that case, the authors can use CODA or CACOA approach to show the significance of the compositional shift. At least a couple of independent sequencing rounds is required to sound convincing. After seeing other data in this manuscript, I do not doubt the main results here, but the field requirements do exist, and I would recommend the authors to follow them. Here are useful links:
https://github.com/kharchenkolab/cacoa
https://www.biorxiv.org/content/10.1101/2022.03.15.484475v1 3. Very often, the authors do not show the embryo development stages in the figures and in the narrative in the text of Results. I find it interfering with my reading, as I always need to go and search if the authors mean neural crest premigratory stage, or immediate postmigratory, or when the derivatives are already formed (for this, every time I navigate to figure legends). This is specifically important when the authors talk about the bridge cells (glial-melanocyte SCP like progenitors) – if those persist long beyond the neural crest migration, it is good to know for how long? In that case, they are rather bipotent melanocyte fate-biased SCPs (broad definition for all glial lineage at this point, including satellite and BCCs). It would be nice if the authors could do RNAscope or HCR to show if they co-express FoxD3, Sox2 and MITF, and also TYR, DCT, PMEL (whatever the authors will choose) at E3 to E6. In case of analyzing the bridge cells in silico, it would be really good if the authors would show the progressing expression of the key melanocyte markers and glial lineage and neural crest TF markers (Sox2, FoxD3, Ets1, AP2, Twist1, Twist2 Snai1, Snai2 etc) along the pseudotime in single cell data in Figure S9 – this figure is nice, and I am pretty happy with it, but I still think that it will benefit from proper trajectory analysis of the bridge with heatmap showing the timeline of transition and key transiting TFs and main markers arranged in a heatmap by pseudotime. I am curious if the authors see ITGA4 and PRDM12 expression in the bridge (see Kastriti and Faure at al., EMBO Journal 2022

© 2024. Published by The Company of Biologists under the terms of the Creative Commons Attribution License (https://creativecommons.org/licenses/by/4.0/).

for satellite/SCP signatures vs neural crest). The authors might also benefit from checking their cell states by using:
https://adameykolab.hifo.meduniwien.ac.at/glia_gene_umap/?fbclid=IwAR2pqUifW-2Pp1t2Non-zrwxZYxMDK-frf8Cg_DgohHlPmCUPZO-GaWQacs This link might be of some help (the link corresponds to Kastriti and Faure et al., EMBO).

4. The RNA Velocity analysis requires the original references to RNA Velocity paper (not only the updated method).

5. The clustering in Figure 5B is of insufficient resolution, as it does not identify mature melanocytes in control condition as a separate cluster (obviously it exists as a well-separated cluster if you plot only control cells). Instead, these melanocytes are mixing with a part of the bridge in the joint embedding. This leads to not so good identification of all proper genes characterizing the bridge and the progression (in that case, the bridge will be much larger/longer as compared to what is shown in 5B). This part can be improved. I respect that the authors show dot plot in Figure 5, but I would prefer to see the expression of MITF, DCT, Sox10, Sox2, FoxD3, Isl1, Phox2b on UMAPs next to panels A and B in the main figure. This would give a reviewer and readers much better feeling about the data.

6. One alternative explanation (still close to author's interpretation) of RA inhibition bridge-melanocyte phenotype is that the SCPs become biased towards melanocyte lineage and generate melanocytes longer and in larger amounts as compared to control, long after NC migration (at E4 and later). This leads to a robust detection of the bridge representing such biased SCPs in experimental condition – strongly supported by Figure S10 (think about bringing it to the main). This angle of interpretation needs to be discussed by the authors in the manuscript. SCPs are known to be located at the DRG surface with morphology different from satellite glial cells, and in mouse embryo, we found them giving rise to a part of dorsal melanocyte population (we never published it, as it was a single collateral result). I am curios if the authors also detect their bridge-to-melanocyte transiting cells at the more ventral branches of sensory and motor nerves. This deserves checking in a more extensive way in control and RA inhibition. Also, the control condition might also contain bridge cells, but at the stage of the analysis, they are left only in very low numbers (the size of the melanocyte cluster in control is really small when it comes to cell numbers, and I am sure that more sequencing will help here to find out if control still contains bridge cells in much lower numbers). I would be curious to see earlier stages (E3.5), also in single cell sequencing. However, I understand that it will require a lot of work and funds, so I think this is not a must. The authors can avoid doing that by implementing some immunohistochemistry or in situ-based techniques. Overall, it is important to understand if the bridge phenotype is totally unnatural/hybrid or it represents temporally-extended SCP biased normal state that dominates the experimental condition with RA inhibition.

7. The authors show single cell plots of the neural crest derivatives, however the autonomic Phox2b+ fates are missing. This is a bit awkward, and I believe that TH/Phox2b sympathetic neuroblasts and symp-biased cells are admixed into the DRG cluster due to their low numbers. This is totally fine, but still better to identify and show those few cells to avoid a confusion among the neural crest community.

8. Line 469 – the authors write about competing interactions between Foxd3 and Mitf, however, Sox2 is also involved into this process – see Adameyko et al., 2012 Development. It would be appropriate to mention this reference as well.

9. Finally, I am curious about detecting the hybrid dorsal neural tube phenotype (Snai/Sox10/Dl1 markers) in single cell data at the level of UMAPs – Figure S6.
I definitely think that a dot plot is insufficient, and the marker overlaps of different subpopulations must be plotted on UMAPs to clearly delineate the hybrid cell types. Where do these hybrid dorsal NT cells reside in the UMAP embedding based on overlapping markers? Using numbers for clusters and cell populations is not intuitive enough. The compositional shifts in Figure S6 could be analyzed properly with more independent sequencing sets done and shown with CACOA, CODA or similar approaches. This should work well for the authors, but this is not a must (in case the authors are limited by their resources).

© 2024. Published by The Company of Biologists under the terms of the Creative Commons Attribution License (https://creativecommons.org/licenses/by/4.0/).

10. Do these hybrid dorsal neural tube cells ever exist in control at earlier stages in lower numbers (I do not think so) prior to border establishment?

Overall, I am looking forward to see the revised version of this manuscript with a great enthusiasm. I am very much interested in this study, and therefore I have many questions and requests. I will understand if the authors will be able to address only a part of it.

Reviewer 2

Advance summary and potential significance to field

This study delves into the influence of dorsal neural tube-derived retinoic acid (RA) on the critical segregation of central and peripheral lineages pivotal for tissue patterning. Through inhibition of retinoic acid activity, the study uncovers co-expression of markers for neural crest, roof plate, and dI1 interneurons, highlighting a failure in proper lineage segregation mediated by Notch activity.

Comments for the author

They discovered a notable difference in gene expression levels compared to embryos exhibiting normal RA activity. Subsequently, they demonstrated that the lack of RA impeded the correct segregation of neural crest (NC), roof plate (RP), and dI1 interneuron fates, leading to the simultaneous expression of lineage markers within individual cells. Lastly, their findings suggest that Notch activity, under the regulation of RA, significantly contributes to the separation process between RP and dI1 interneurons.

These findings elucidate the intricate molecular mechanism underlying dorsal neural fate segregation during development, shedding crucial light on cell specification within these lineages, which is essential for understanding the complex and adaptable nature of developmental processes. Altogether, this article is nicely performed and nicely written. They performed a scRNA studies and validated some of their results either at the mRNA or protein levels or using some transient transfection assays. Overall, I do not have major criticisms.

The researchers conducted a significant co-electroporation experiment involving Msx1-Citrine labeling with either control or RARa403 plasmid on (2*10) E2.5 quail embryos, followed by the isolation of mRNA from Citrine-positive cells at E4. An important question arises: do Citrine-positive cells accurately and exclusively represent the specific cells they are intended to label, considering the molecular-level analysis? The authors should delve deeper into this aspect in their discussion.

Minor points:
* In the title, authors should use "roof plate" instead of "RP" for clarity and to ensure understanding for any reader. This explicit terminology enhances comprehension and avoids potential confusion regarding the subject matter.

* The authors should specify in the title or the abstract that these studies were performed in quail. This is of importance since we know that there are differences between birds and mammals.

* At the end of the legend of figure 2, it is written N=8,8 embryos for each treatment, I am not sure I understand “8,8” since they refer to 2*10 somewhere else.

Reviewer 3

Advance summary and potential significance to field

This well-done study examines the requirement for retinoic acid signaling in dorsal neural tube development. A previous manuscript from the same laboratory demonstrated that a dominant negative RAR expression in the dorsal neural tube alters NC, interneurons, and RP cell fates, generating cells expressing markers of mixed, more progenitor-like fates. In that study, the temporal and spatial segregation of dorsal neural tube lineages were important as inhibition of RA

© 2024. Published by The Company of Biologists under the terms of the Creative Commons Attribution License (https://creativecommons.org/licenses/by/4.0/).

signaling resulted in the extension of NC emigration into the time when the RP should be developed, which disrupts adjacent interneuron development. In another previous study from the lab, the role of Notch signaling in the RP/interneuron fate decision.

In this study, the authors have successfully applied the scRNAseq approach to analyze the same experimental manipulations. The bioinformatic analysis is outstanding and is validated by manipulations in the quail system. The detailed analysis provides a deeper understanding of the abnormal cells of mixed fates generated. Importantly, these results confirm and extend the previous findings, enhancing our comprehension of retinoic acid signaling in dorsal neural tube development into multiple lineages.

Comments for the author

However, the specific advances and why a deeper dive using single-cell approaches is necessary should be better articulated in the abstract and introduction. This is particularly important as many of this manuscript's conclusions were already known from previous studies from the Kalcheim laboratory.

Specific comments

Fig 1 A-D is too small. I suggest deleting panel D, which is particularly small and likely unnecessary.

Fig 2 L – controls should be shown too

How is anterior-posterior patterning of the neural tube affected by Rara403 expression?

Methods for determining the function of differentially expressed genes were not described. Was GO analysis done? What database was used?

On pg 5, line 114, while dorsal fates were enriched, ventral subtypes were also found, and this is attributed to leaky expression of the Msx1-citrine. However, this could also be an artifact of sorting. Including Citrine in the dotblot analysis could distinguish between these possibilities.

Some important points are being made with data in the supplemental figures that are not represented in the main figures. Consider making all the major points in the main figures and moving some of the supporting. data into the supplemental.

First revision

<u>Author response to reviewers' comments</u>

MS ID#: DEVELOP/2024/202973
MS TITLE: Retinoic acid, an essential component of the RP organizer, promotes the spatio-temporal segregation of dorsal neural fates
AUTHORS: Dina Rekler, Shai Ofek, Sarah Kagan, Gilgi Friedlander, and Chaya Kalcheim

Reviewer 1 Advance Summary and Potential Significance to Field:

The authors investigate the role of retinoic acid (RA) in the formation and differentiation of cell types in the developing neural tube, focusing particularly on the segregation of neural crest cells, roof plate and the rest of the dorsal central nervous system cells. The authors employed in-ovo gene misexpression and single-cell RNA profiling to explore how RA influences the spatial and temporal segregation of cell fates. The findings indicate that RA is crucial for the correct separation between NC and dorsal CNS cells forming the roof plate (RP). The study reveals that RA might modulate this process by suppressing BMP and Wnt signaling pathways, which are essential for the initiation of the epithelial-mesenchymal transition in NC cells. The observation of a hybrid

© 2024. Published by The Company of Biologists under the terms of the Creative Commons Attribution License (https://creativecommons.org/licenses/by/4.0/).

cell phenotype in the dorsal neural tube in case of RA inhibitor treatment is the pivotal observation, which is most interesting to me. Indeed, the authors convincingly show that the absence of RA signaling disrupts this organized segregation, leading to an intriguing mixing of cell type features, also including the invasion of RP by dI1 interneurons, which normally should not occur. The study also identifies that Notch signaling, essential for RP and dI1 interneuron formation, is restricted by RA to the boundary regions between these cells, facilitating proper spatial separation.
The last but not the least, the authors show that RA signaling influences the processes of fate selection in the neural crest lineage leading to the expansion of melanocyte-glial progenitors, most likely Schwann cell precursors and satellite glial cells that become biased towards melanocyte lineage in the absence of RA signaling. This is the second most interesting results to me.
Overall, the study is well done and is technically sound in a general way, though it requires some technical tuning prior to publishing.

Reviewer 1 Comments for the Author:
Overall, the study is well done and is technically sound in a general way, though it requires some technical tuning prior to publishing.
REPLY: We thank this referee for their positive assessment of our study. We now addressed a significant number of the questions raised to the best of our ability. We hope this answers to a large extent the referee's comments. Below, please find a point-by-point explanation.

1. The single cell data require the inclusion of a technical supplementary figure showing quality controls such as numbers of genes per cell, numbers of reads per cell, doublet detection and removal, mitochondrial fraction, top 20 genes per every cluster (unbiasedly define) for every umap embedding.
REPLY- As requested, a figure with quality controls was added and the top 20 genes per UMAP appear now as Supplementary Tables 1,2, and 3.

2. *Figure 1E,F: When it comes to showing compositional differences between control and experimental condition (RA inhibition), the current standards in the filed suggest doing several sequencing rounds (3-4 per condition, could be smaller batches) to calculate the significance of the effect, as single cell datasets are prone to dropouts and other technical problems. In that case, the authors can use CODA or CACOA approach to show the significance of the compositional shift. At least a couple of independent sequencing rounds is required to sound convincing. After seeing other data in this manuscript, I do not doubt the main results here, but the field requirements do exist, and I would recommend the authors to follow them. Here are useful links:*
https://github.com/kharchenkolab/cacoa
https://www.biorxiv.org/content/10.1101/2022.03.15.484475v1

REPLY- We are pleased to read that this reviewer repeatedly mentions that they trust the data. Nevertheless, they suggest we perform additional scRNA sequencing with several replicates per experimental point as a "standard procedure," effectively asking us to repeat the entire experimental paradigm. We understand the referee's request and wish we could have addressed this issue at the project's onset. Unfortunately, due to technical limitations, such as funding for such an expensive task, we could not perform triplicates or more repetitions per experimental treatment. Currently, due to the above reason and also to time constraints of the review process, we cannot provide additional rounds of sequencing. While we agree that duplicate samples should become the standard, a survey of various accomplished scRNA seq articles shows that some studies use duplicates or triplicates, while others do not. See for example: a) Faure, L., et al (2020). Nature Communications. b) Celotto, et al (2023). Elife. c) Farra, et al. (2024). Aging Cell. d) Zhao, et al (2024) bioRxiv. e) Negretti, et al (2021). Development. F) Doda, et al. (2023). Development.

Notably, none of the other two reviewers expressed any concern about this matter.

Nonetheless, we are confident that our analysis faithfully represents the biological reality: a) The number of embryos pooled per experimental treatment is significant (N=10/treatment). b) The resulting gene profiles are highly reliable and consistent with published single-cell data. Many of

© 2024. Published by The Company of Biologists under the terms of the Creative Commons Attribution License (https://creativecommons.org/licenses/by/4.0/).

the RNA profiles are consistent with our previously reported data, thus strengthening the grounds on which this project stands, and providing a basis for addressing the novel questions examined in this study. c) Most importantly, based on the sequencing results, we performed extensive validations through ISH, immunohistochemistry, reporter analysis, and most importantly, functional experiments, all of which consistently support the soundness of our scRNA seq.

3. *Very often, the authors do not show the embryo development stages in the figures and in the narrative in the text of Results. I find it interfering with my reading, as I always need to go and search if the authors mean neural crest* premigratory stage, or immediate postmigratory, or when the derivatives are *already formed (for this, every time I navigate to figure legends). This is specifically important* when the authors talk about the bridge cells (glial-melanocyte SCP like progenitors)
REPLY- Embryo stages were now added to the text. They all appear in the respective legends to make reading easier, we did not realize that it would make it more difficult…). Essentially, there are two relevant stages in this paper, the NC stage is referred to as E2.5 and the RP stage is E4. Now, based on this referee's request, we added older stages (E5, E6) to an experiment and these are detailed in both the text and legends to the respective figures.

……This is specifically important when the authors talk about the bridge cells (glial- melanocyte SCP like progenitors) – if those persist long beyond the neural crest migration, it is good to know for how long? In that case, they are rather bipotent melanocyte fate-biased SCPs (broad definition for all glial lineage at this point, including satellite and BCCs). It would be nice if the authors could do RNAscope or HCR to show if they co-express FoxD3, Sox2 and MITF, and also TYR, DCT, PMEL (whatever the authors will choose) at E3 to E6.
REPLY- *Unfortunately, RNAscope or HCR were not reliable for visualizing gene co-expression in such small and scattered populations. Hence, to meet the referee's request as to the temporal dynamics of bridge cells, we performed ISH for 3 markers: C1QTNF3 as a unique bridge marker present only under treated conditions, SERAF for glia, and EDNRB2 for melanocytes. Analysis at E3, as suggested, is rather impractical as normal crest cells still emigrate and this is pretty close to the time of electroporation; therefore we performed ISH at E5 and E6, as a follow up of our E4 data.*
Indeed, in addition to the control patterns of expression of each of the above genes, we clearly documented at E5 all the 3 transcripts in ectopic sites under treated conditions. For instance, C1QTNF3 (bridge) was expressed in mesenchyme dorsal to the neural tube, SERAF (glia) was also present in dermis and epidermis, as expected for pigment cells. Reciprocally, EDRNB2 (melanocytes) was also expressed in the mesenchyme close to the tube, as well as along nerve fibers, a trait typical of Schwann cell progenitors. At E6, however, no differences were apparent any longer, suggesting that bridge progenitors persist for at least two days following the completion of NC production under otherwise control conditions. These data were added as Supplementary figures S10, S11 and S12.

In case of analyzing the bridge cells in silico, it would be really good if the authors would show the **progressing expression** of the key melanocyte markers and glial lineage and neural crest TF markers (Sox2, FoxD3, Ets1, AP2, Twist1, Twist2, Snai1, Snai2 etc) **along the pseudotime in single cell data in Figure S9** – this figure is nice, and I am pretty happy with it, but I still think that it will **benefit from proper trajectory analysis of the bridge with heatmap showing the timeline of transition and key transiting TFs and main markers arranged in a heatmap by pseudotime.**
REPLY- A heatmap illustrating the trajectory of the relevant genes along the pseudotime (pDRG, bridge, melanocytes) was now added to complement original Fig S9, as suggested. These data appear in new figure 7.

I am curious if the authors see ITGA4 and PRDM12 expression in the bridge (see Kastriti and Faure at al., EMBO Journal 2022 for satellite/SCP signatures vs neural crest). The authors might also benefit from checking their cell states by using:
https://adameykolab.hifo.meduniwien.ac.at/glia_gene_umap/?fbclid=IwAR2pqUifW- 2Pp1t2Non-zrwxZYxMDK-frf8Cg_DgohHlPmCUPZO-GaWQacs
This link might be of some help (the link corresponds to Kastriti and Faure et al., EMBO).

© 2024. Published by The Company of Biologists under the terms of the Creative Commons Attribution License (https://creativecommons.org/licenses/by/4.0/).

REPLY- ITGA4 indeed marks the bridge under treated conditions, it is, however, very weak in controls. PRDM12 (which appeared in original Fig S7) mainly characterizes the pDRG subset in both control and treated conditions. The dynamics of both genes along the pseudotime was now added to the heatmap presented in Fig. 7 and the reference by Kastriti et al cited in the text.
We also appreciate the recommendation to utilize the pagoda2 package outlined by Kastriti and Adameyko. This suggestion is valuable, and we are considering incorporating it into our ongoing research. Specifically, we may apply this approach in our upcoming analyses, which focus on exploring additional cell types within the dorsal tube.

4. The RNA Velocity analysis requires the original references to RNA Velocity paper (not only the updated method).
The original paper by La Manno (2018) which laid the theoretical foundation and provided the first practical implementation for estimating RNA velocity from scRNA data, was cited in the original version along with that of Bergen et al (2020).

5. The clustering in Figure 5B is of insufficient resolution, as it does not identify mature melanocytes in control condition as a separate cluster (obviously it exists as a well-separated cluster if you plot only control cells). Instead, these melanocytes are mixing with a part of the bridge in the joint embedding. This leads to not so good identification of all proper genes characterizing the bridge and the progression (in that case, the bridge will be much larger/longer as compared to what is shown in 5B). This part can be improved. I respect that the authors show dot plot in Figure 5, but I would prefer to see the expression of MITF, DCT, Sox10, Sox2, FoxD3, Isl1, Phox2b on UMAPs next to panels A and B in the main figure. This would give a reviewer and readers much better feeling about the data.
REPLY- The initial small size of the melanocyte cluster is a result of the electroporation timing. Given this limited starting population, we have concerns that additional subclustering could produce even smaller cell groups, potentially compromising the reliability of our analysis. As requested, we have now included a series of UMAPs showcasing the suggested genes, along with a few additional ones. However, it's worth noting that PHOX2B is not present in our transcriptome (please refer to our response to item #7 for more details). These data are presented in Figure S8.

6. One alternative explanation (still close to author's interpretation) of RA inhibition bridge-melanocyte phenotype is that the SCPs become biased towards melanocyte lineage and generate melanocytes longer and in larger amounts as compared to control, long after NC migration (at E4 and later). This leads to a robust detection of the bridge representing such biased SCPs in experimental condition – strongly supported by Figure S10 (think about bringing it to the main). This angle of interpretation needs to be discussed by the authors in the manuscript.
REPLY- This idea was discussed, as suggested, and Fig S10 was brought to the main section as Fig.10.

SCPs are known to be located at the DRG surface with morphology different from satellite glial cells, and in mouse embryo, we found them giving rise to a part of dorsal melanocyte population (we never published it, as it was a single collateral result).
There is no evidence in avians for expression of Schwann cell markers in the DRG periphery. For example, the referee may look at the pattern of SERAF and KNDC1 expression in control embryos where they are mainly restricted to peripheral nerves (this study). Having said that, earlier markers such as Sox10 and Foxd3 are expressed in the periphery of DRGs where they may behave as both satellite glia and melanocyte progenitors, consistent with the referee's observation. To note, however, is that neither of these markers is known for its unique selectivity to SCPs.

I am curios if the authors also detect their bridge-to-melanocyte transiting cells at the more ventral branches of sensory and motor nerves. This deserves checking in a more extensive way in control and RA inhibition.
REPLY- We assume the referee asks this question because it is in the distal regions of peripheral nerves where the SCPs leave the nerve and generate melanocytes. We do not have an answer to

© 2024. Published by The Company of Biologists under the terms of the Creative Commons Attribution License (https://creativecommons.org/licenses/by/4.0/).

this question. This would require growing embryos to relatively late stages when the delamination of SCPs from the nerves takes place. In principle, we expect this to occur both in controls and in RARa403-treated conditions, hence it will be difficult to detect a difference. In particular, regarding the identity of these cells, our attempts to double hybridize single scattered cells for GM markers, were not successful.

--Also, the control condition might also contain bridge cells, but at the stage of the analysis, they are left only in very low numbers (the size of the melanocyte cluster in control is really small when it comes to cell numbers, and I am sure that more sequencing will help here to find out if control still contains bridge cells in much lower numbers). I would be curious to see earlier stages (E3.5), also in single cell sequencing. However, I understand that it will require a lot of work and funds, so I think this is not a must. The authors can avoid doing that by implementing some immunohistochemistry or in situ-based techniques. Overall, it is important to understand if the bridge phenotype is totally unnatural/hybrid or it represents temporally-extended SCP biased normal state that dominates the experimental condition with RA inhibition.

REPLY- The referee raises an excellent question and we alluded to it at the end of the original Discussion. In principle, we would argue that progenitors with the capacity to generate both glia cell types and melanocytes are part of a natural developmental trajectory, prior to fate segregation, and not only a result of RA inhibition.
However, this does not mean that under natural conditions, the alternative fates are co-expressed in single cells. For example, do SCPs sitting along the nerve co-express melanocyte traits, or chromaffin markers, etc? It may be only upon detachment from the nerve, migration to the appropriate anlagen, etc, that these bi-fated (or multi-fated) progenitors express their full capacities. Under the latter conditions, cells would lose the SCP phenotype and gain, for example, a melanocyte trait, making it difficult to unravel double markers in the same cell. Alternatively, it is possible that these progenitors express extremely low levels of the alternative fate, making it very difficult to detect experimentally. We believe that inhibiting RA signaling was an opportunity to uncover the double progenitor state.

Having said that, even if we wished to engage in this experiment, demonstration is technically very difficult because:
1- The peripheral, NC-derived cells are a minority of the cells collected during the process towards the transcriptome analysis, and this is simply because we manually dissected the neural tubes along with a "contamination" of paraxial mesoderm that contained the NC-derived progenitors. Therefore, performing additional sequencing analyses using the same technique will not improve this fact, and melanocytes, etc. will still remain as significant, yet small clusters.

2- At E4 in controls, no bridge cells are detected. Performing an earlier analysis as suggested, e.g; E3.5, is at the limit between normal melanocyte emigration from the tube (the direct NC-derived melanocytes which are the last wave of emigrating cells, see Krispin et al, Development) and the end of NC production. Such an accuracy cannot be reached experimentally in a faithful manner due to the fast temporal dynamics.

3- Performing immunohistochemistry/ISH at various stages of normal development could be a possible approach. The existence of bridge cells under normal conditions would be resolved only if we succeed in detecting cells that co-express glial and melanocyte markers. As mentioned in reply to a precedent item, we did not succeed with HCR or RNAscope to discriminate gene co-expression in scattered cells. Immunohistochemistry would have been a solution, yet suitable antibodies that cross-react with avian tissue and fulfill our needs are not available (we tried several with no success). Addressing the mouse embryo could be a good alternative for this, but is beyond the scope of the present study. We are nevertheless aware of the significance of this subject. Approaching it would require a different experimental protocol provided this can be demonstrated experimentally, as mentioned above.

7. The authors show single cell plots of the neural crest derivatives, however, the autonomic Phox2b+ fates are missing. This is a bit awkward, and I believe that TH/Phox2b sympathetic neuroblasts and symp-biased cells are admixed into the DRG cluster due to their low numbers. This is totally fine, but still better *to identify and show those few cells to avoid a confusion among the*

© 2024. Published by The Company of Biologists under the terms of the Creative Commons Attribution License (https://creativecommons.org/licenses/by/4.0/).

neural crest community.
REPLY- Electroporation to the NT was performed at a stage that follows emigration of sympathoblasts, the first progenitors to leave the neural primordium. Hence, they are not represented in our data. A sentence regarding this issue appears in the original results section. Furthermore, careful examination of autonomic genes such a tyrosine hydroxylase and Phox2B clearly shows they are absent from the transcriptome. Finally, it is long known that in mice, DRG harbor some sympathetic cells as well, however, this is not the case in avians.

8. Line 469 – the authors write about competing interactions between Foxd3 and Mitf, however, Sox2 is also involved into this process – see Adameyko et al., 2012 Development. It would be appropriate to mention this reference as well.
REPLY- We added a sentence on Sox2-MITF competing interactions in the mouse and cited the reference accordingly, thanks.

9. Finally, I am curious about detecting the hybrid dorsal neural tube phenotype (Snai/Sox10/Dl1 markers) in single cell data at the level of UMAPs – Figure S6. I definitely think that a dot plot is insufficient, and the marker overlaps of different subpopulations must be plotted on UMAPs to clearly delineate the hybrid cell types. Where do these hybrid dorsal NT cells reside in the UMAP embedding based on overlapping markers? Using numbers for clusters and cell populations is not intuitive enough. The compositional shifts in Figure S6 could be analyzed properly with more independent sequencing sets done and shown with CACOA, CODA or similar approaches. This should work well for the authors, but this is not a must (in case the authors are limited by their resources).
REPLY- We believe that the overlaps requested at the UMAP level appear in original Fig 3A, which now became 4A.

10. Do these hybrid dorsal neural tube cells ever exist in control at earlier stages in lower numbers (I do not think so) prior to border establishment?
REPLY- Most likely not. At earlier stages, no RP or dI1-specific genes are detected in the dorsal tube. There appears to be a clear temporal segregation between earlier NC and later RP, and at the latter time point, a clear spatial separation between dorsal RP and ventrally adjacent dI1 cells.

Overall, I am looking forward to see the revised version of this manuscript with a great enthusiasm. I am very much interested in this study, and therefore I have many questions and requests. I will understand if the authors will be able to address only a part of it.
We thank again this referee for detailed insights on our study. We revised the manuscript to the best of our ability and hope to have answered at least part of the questions raised.

****** Reviewer 2 Advance Summary and Potential Significance to Field:*
This study delves into the influence of dorsal neural tube-derived retinoic acid (RA) on the critical segregation of central and peripheral lineages pivotal for tissue patterning. Through inhibition of retinoic acid activity, the study uncovers co-expression of markers for neural crest, roof plate, and dI1 interneurons, highlighting a failure in proper lineage segregation mediated by Notch activity.

Reviewer 2 Comments for the Author:
They discovered a notable difference in gene expression levels compared to embryos exhibiting normal RA activity. Subsequently, they demonstrated that the lack of RA impeded the correct segregation of neural crest (NC), roof plate (RP), and dI1 interneuron fates, leading to the simultaneous expression of lineage markers within individual cells. Lastly, their findings suggest that Notch activity, under the regulation of RA, significantly contributes to the separation process between RP and dI1 interneurons.
These findings elucidate the intricate molecular mechanism underlying dorsal neural fate segregation during development, shedding crucial light on cell specification within these lineages, which is essential for understanding the complex and adaptable nature of developmental

© 2024. Published by The Company of Biologists under the terms of the Creative Commons Attribution License (https://creativecommons.org/licenses/by/4.0/).

processes. Altogether, this article is nicely performed and nicely written. They performed a scRNA studies and validated some of their results either at the mRNA or protein levels or using some transient transfection assays. ***Overall, I do not have major criticisms.***
REPLY- We thank this reviewer for their positive evaluation of our study and address below each of the questions raised.

*The researchers conducted a significant co-electroporation experiment involving Msx1-Citrine labeling with either control or RARa403 plasmid on (2*10) E2.5 quail embryos, followed by the isolation of mRNA from Citrine-positive cells at E4. An important question arises: do Citrine-positive cells accurately and exclusively represent the specific cells they are intended to label, considering the molecular-level analysis? The authors should delve deeper into this aspect in their discussion.*
REPLY- MSX1-citrine was implemented to enrich for dorsal cell types but it is not meant to be a specific marker for a given cell type. As apparent in Fig.1 B,C, electroporation attained mainly the dorsal half of the neural tube, thus, resulting in a significant enrichment of dorsal cell types with a minimal leakage to more ventral cell types. Furthermore, no differences in the electroporation patterns between controls and RARa403-treated neural tubes was monitored. This is also consistent with the bioinformatic data which detected dorsal NC derivatives, roof plate cells and dorsal interneurons of various types, as expected. Nevertheless, a small cluster of putative motoneurons was also evidenced in the analysis, but no floor plate cells or ventral interneuron types. As the referee might have noticed, this did not interfere at all with the reliability of the RNA seq. This was explained in the Results section under "Assignment of transcriptomes to cell identities".

Minor points:
*   *In the title, authors should use "roof plate" instead of "RP" for clarity and to ensure understanding for any reader. This explicit terminology enhances comprehension and avoids potential confusion regarding the subject matter.*
REPLY- Corrected, thanks

*   *The authors should specify in the title or the abstract that these studies were performed in quail. This is of importance since we know that there are differences between birds and mammals.*
REPLY- This was now added to the abstract section.

*   *At the end of the legend of figure 2, it is written N=8,8 embryos for each treatment, I am not sure I understand "8,8" since they refer to 2*10 somewhere else.*
*REPLY- Eight embryos per treatment refers to the specific experiment quantified in Fig. 2M that examined axonal projections (respective images in 2L,L'). The 2*10 number the reviewer mentions is the number of embryos electroporated with either control or RARa403 (10/treatment) that served for the RNA seq. This was clearly explained in the respective places.*

****** Reviewer 3 Advance Summary and Potential Significance to Field:*
This well-done study examines the requirement for retinoic acid signaling in dorsal neural tube development. A previous manuscript from the same laboratory demonstrated that a dominant negative RAR expression in the dorsal neural tube alters NC, interneurons, and RP cell fates, generating cells expressing markers of mixed, more progenitor-like fates. In that study, the temporal and spatial segregation of dorsal neural tube lineages were important as inhibition of RA signaling resulted in the extension of NC emigration into the time when the RP should be developed, which disrupts adjacent interneuron development. In another previous study from the lab, the role of Notch signaling in the RP/interneuron fate decision.
In this study, the authors have successfully applied the scRNAseq approach to analyze the same experimental manipulations. The bioinformatic analysis is outstanding and is validated by manipulations in the quail system. The detailed analysis provides a deeper understanding of the abnormal cells of mixed fates generated. Importantly, these results confirm and extend the previous findings, enhancing our comprehension of retinoic acid signaling in dorsal neural tube development into multiple lineages.
REPLY- We thank this reviewer for their positive evaluation of our study and address below each of the questions raised to the best of our ability.

© 2024. Published by The Company of Biologists under the terms of the Creative Commons Attribution License (https://creativecommons.org/licenses/by/4.0/).

---- Reviewer 3 Comments for the Author:
However, the specific advances and why a deeper dive using single-cell approaches is necessary should be better articulated in the abstract and introduction. This is particularly important as many of this manuscript's conclusions were already known from previous studies from the Kalcheim laboratory.
REPLY- We added sentences to the abstract and introduction to better explain why a single cell approach was necessary. In fact, without a scRNA seq, we would not be able to demonstrate the role of RA signaling in separation of NC, RP and dI1 lineages in the dorsal NT, or between glia and melanocyte progenitors in the PNS. Thanks.

Specific comments

1- Fig 1 A-D is too small. I suggest deleting panel D, which is particularly small and likely unnecessary.
REPLY-We have corrected the panels, as requested.

2- Fig 2 L – controls should be shown too
REPLY- As requested by the referee, a control image is now shown as panel L, along with an image of the treated NTs (panel M).

3- *How is anterior-posterior patterning of the neural tube affected by Rara403 expression?*
REPLY- At early stages, countergradients of RA and FGF8 emanating from the paraxial mesoderm are indeed involved in rostro-caudal patterning. Furthermore, in the early neural tube, high RA levels posteriorize the anterior regions, causing anterior structures like the forebrain and midbrain to develop characteristics of more posterior regions like the hindbrain and spinal cord, an effect mediated by RA's effects on Hox gene expression. In the trunk, it is known that RA mildly influences the expression strength of Hox genes like hoxb1, hoxb2, hoxb4, hoxb8, and hoxa1 during later stages, suggesting a role in maintaining the A-P pattern in the trunk. To note is that our experiments address RA signaling stemming exclusively from the dorsal neural tube but do not affect mesoderm-derived RA. In addition, our experiments attained a very restricted area of the axis (somite levels 20-25, the flank region), and were undertaken at a stage following initial rostro-caudal patterning of the neural primordium. Consistently, no significant effect was apparent in the levels of expression of hoxb1, hoxb2, hoxb4, hoxb8, or hoxa1 between control and RA-deprived cases, indicative of no effect on A-P patterning under the present conditions.

4- *Methods for determining the function of differentially expressed genes were not described. Was GO analysis done? What database was used?*
REPLY- Our analysis began by utilizing several databases (e.g. Ingenuity Pathway Analysis, Gene Ontology etc.). However, we soon realized these were insufficient for our needs, as they do not address relevant developmental processes, and also lacked many of the genes of interest identified in the transcriptome analysis. Consequently, we embarked on a lengthy yet productive and revealing manual survey. This involved extensive literature searches combined with our existing knowledge to select, from among the significantly changed genes, those likely to have functional importance.

5- *On pg 5, line 114, while dorsal fates were enriched, ventral subtypes were also found, and this is attributed to leaky expression of the Msx1-citrine. However, this could also be an artifact of sorting. Including Citrine in the dotblot analysis could distinguish between these possibilities.*
REPLY- Fig1 panels B and C show few cells in both the control Msx1-citrine and in the RARa403/Msx1-citrine, respectively, located in the ventral half of the neural tube, consistent with the bioinformatic data. Nevertheless, the significant electroporation attained the dorsal region of the tube, as expected. Hence, this is not likely to result from a sorting artifact. We know from previous experience unrelated to this study, that several reporter plasmids show a leaky behavior at early stages that resolves later on.

6- Some important points are being made with data in the supplemental figures that are not represented in the main figures. Consider making all the major points in the main figures and moving some of the supporting. data into the supplemental.

© 2024. Published by The Company of Biologists under the terms of the Creative Commons Attribution License (https://creativecommons.org/licenses/by/4.0/).

REPLY: As a result of the revision process, 6 figures and 3 supplemental tables were added. The manuscript now contains 11 main figures (with permission of the editorial), 16 supplemental ones and 5 Supplemental Tables. As requested by this referee, 3 figures were moved from the Supplemental to the main section and a new figure was added to the main figures section.

Second decision letter

MS ID#: DEVELOP/2024/202973

MS TITLE: Retinoic acid, an essential component of the roof plate organizer, promotes the spatio-temporal segregation of dorsal neural fates

AUTHORS: Dina Rekler, Shai Ofek, Sarah Kagan, Gilgi Friedlander, and Chaya Kalcheim
ARTICLE TYPE: Research Article

I am happy to tell you that your manuscript has been accepted for publication in Development, pending our standard publication integrity checks.

Reviewer 1

Advance summary and potential significance to field

The authors significantly improved the manuscript during the revision stage by adding quality controls, better trajectory analysis and some important clarifications in the text. The resulting manuscript convincingly demonstrates the role of RA signaling in dorsal neural tube stratification and neural crest gradual fate restriction.

Comments for the author

I am happy with the current revision and hope to see the paper soon in print. I thank the authors for all the improvements.

© 2024. Published by The Company of Biologists under the terms of the Creative Commons Attribution License (https://creativecommons.org/licenses/by/4.0/).
